# Impact of Proline-Rich Salivary Proteins on Interactions between Catechin and Polysaccharides

**DOI:** 10.1021/acs.jafc.6c07975

**Published:** 2026-08-28

**Authors:** Philippe Rodrigues Benedetti, Leociley Rocha Alencar Menezes, Guilherme Lanzi Sassaki

**Affiliations:** Department of Biochemistry and Molecular Biology, Federal University of Paraná, Curitiba, Paraná 81.531-980, Brazil

**Keywords:** STD NMR, epitope map, dissociation constant, mannan, pectin

## Abstract

The modulation of interactions between catechin and two structurally distinct wine polysaccharides (mannan and pectin) by proline-rich salivary proteins (PRPs) was investigated using saturation transfer difference nuclear magnetic resonance (STD NMR). Mannan–catechin interactions, driven by CH-π stacking and hydrophobic effects, were weakened by both acidic and basic PRPs. This competitive interference increased the apparent dissociation constant (*K*_D_) from ≈231 μM to ≈272 and 279 μM, respectively. Conversely, catechin–pectin binding (baseline *K*_D_ ≈ 230 μM) was unaffected by acidic PRPs but synergistically enhanced by basic PRPs (*K*_D_ ≈ 195 μM) due to favorable electrostatic attractions. Epitope mapping confirmed that catechin binds differently depending on the polysaccharide structure. Ultimately, the results reveal a dual regulatory pattern: PRPs act as competitive antagonists for neutral polysaccharides (mannan) and cooperative synergists for charged ones (pectin). These molecular insights provide a concrete biophysical basis for strategically managing wine astringency and sensory quality.

## Graphical abstract

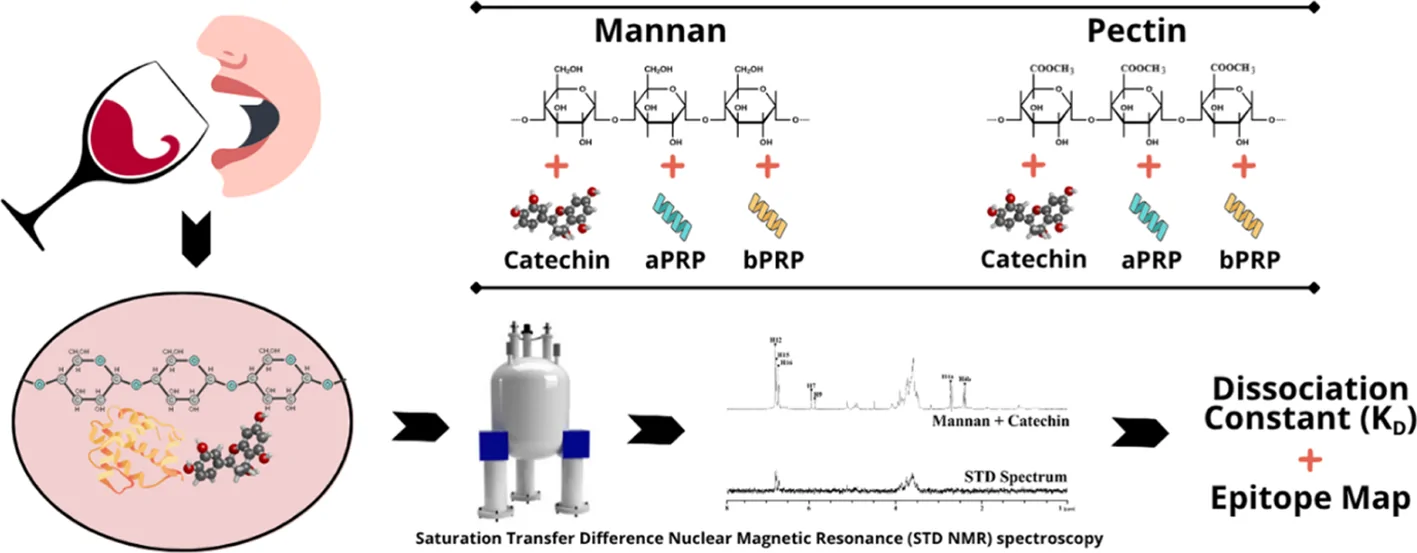

## 1 Introduction

The chemical complexity and flavor diversity of wine result from various factors, such as grape variety, vineyard edaphoclimatic conditions, and the winemaking process.^1,2^ These elements influence wine composition, which can be adjusted according to the characteristics desired by the winemaker. There are thousands of grape varieties worldwide, most of which belong to the species *Vitis vinifera*. Among them, Merlot stands out for its adaptability and distinctive characteristics of the wines it produces.^3^

Wine is primarily composed of water (86%) and ethanol (12%), along with glycerol and polysaccharides (1%), organic acids (0.4%), polyphenols (0.1%), minerals, and other compounds (0.5%). Glycerol and polysaccharides, ranging from 4 to 10 g/L, are the main compounds after water and ethanol, significantly contributing to wine quality.^4,5^ Polysaccharides originate from the cell walls of grapes and yeasts and are extracted during winemaking.^6^ Their effects on wine depend on their size, composition, molecular structure, and origin. They are classified into three main groups: (I) polysaccharides rich in arabinose and galactose, (II) polysaccharides rich in rhamnogalacturonans (RG-I and RG-II) from grape cell walls, and (III) mannoproteins and mannans released by yeasts during fermentation.^7,8^

Phenolic compounds are essential for wine quality, despite accounting for only 0.1% of its composition.^9^ Among them, flavonoids have a structure consisting of two aromatic rings connected by a three-carbon chain (C6–C3–C6). Flavanols, a subclass of flavonoids, include catechin and its epimer, epicatechin. In white wines, catechin is the most abundant flavonoid, contributing to their characteristic flavor.^10^ Catechin derivatives have also been identified in grapes and wines.^11,12^

Salivary proteins are divided into several families, with proline-rich proteins being the most important in interactions with phenolic compounds.^13^ They are further subdivided into acidic, basic, and glycosylated PRPs.^14^ Basic PRPs, known for their high affinity for tannins, are the most studied. Besides binding to tannins and preventing their deleterious effects on the gastrointestinal tract, they do not have other specific functional roles.^15,16^ However, *in vitro* and *in vivo* studies show that polyphenols interact more strongly with acidic PRPs than with basic ones.^17,18^ Astringency, the sensation of dryness and constriction of the oral mucosa caused by the interaction between polyphenols and proteins, appears to correlate more strongly with the strength of this interaction with the ability of polyphenols to precipitate proteins.^19^

Wine polysaccharides interact with polyphenols and salivary proteins, but due to their complexity, not all behave the same way.^20^ Their physicochemical properties determine these interactions. In pectins, for example, interactions are stronger with arabinogalactan side chains than with rhamnogalacturonans.^21–23^ These interactions also depend on the protein composition of saliva, which varies according to human genetics.^24^ Thus, polysaccharides can modulate wine astringency, acting as refiners or stabilizers to enhance stability and organoleptic properties.^24,25^ They can modify astringent wines and aid in the production of wines with specific characteristics.^26^ However, further research is needed to fully understand the complexity of the wine matrix and its components, highlighting the importance of balancing the composition and quantity of molecular components to optimize the sensory properties of the final wine.^27^

Nuclear magnetic resonance (NMR) is a powerful technique for investigating interactions between macromolecules and ligands, providing structural and quantitative information about these interactions.^28–30^ In this context, the present study employed NMR to investigate the influence of salivary proteins on the interactions between polysaccharides—extracted from wine and grapes—and catechin, with the aim of providing a comprehensive understanding of the underlying binding mechanisms.

Therefore, this study hypothesizes that acidic and basic PRPs, due to their distinct physicochemical properties, differentially modulate the interactions between catechin and wine polysaccharides, specifically mannan and pectin, by influencing the binding affinity and the underlying mechanisms. We propose that acidic and basic PRPs exert distinct effects on the catechin-polysaccharide interaction, depending on molecular features of the polysaccharides, such as their charge.

## 2 Materials and Methods

### 2.1 Chemicals and Reagents

Catechin (≥98%, HPLC, Sigma-Aldrich, C1251) was used for interaction and STD-NMR binding assays. Deuterium oxide (D_2_O, ≥99.9%, Cambridge Isotope Laboratories) was used as the solvent for NMR experiments. For methylation and GC–MS structural analyses, the following reagents were employed: iodomethane (CH_3_I, ≥99%, Sigma-Aldrich), dimethyl sulfoxide (DMSO, ≥99.9%, Sigma-Aldrich), sodium borodeuteride (NaBD_4_, ≥95%, Sigma-Aldrich), acetic anhydride (≥99%), and pyridine (≥99.5%, anhydrous, Sigma-Aldrich). Chloroform (≥99%) and *meta*-hydroxybiphenyl (≥98%, Sigma-Aldrich) were also used for extraction and uronic acid quantification, respectively. For MALDI-ToF MS, sinapinic acid (≥99%) was used as the matrix. Analytical-grade reagents included trifluoroacetic acid (TFA, ≥99%, Sigma-Aldrich) for salivary protein precipitation and protease inhibition, sodium borohydride (NaBH_4_, ≥98%), potassium hydroxide (KOH), copper­(II) sulfate (CuSO_4_), sodium potassium tartrate, sodium nitrate (NaNO_3_), sodium azide (NaN_3_), and *Tris*–HCl and NaCl for buffer preparation. All reagents were used without further purification and handled according to the protocols described for each analytical technique.

### 2.2 Extraction and Identification of Polysaccharides from Wine and Grapes

To extract polysaccharides from Merlot wine, 750 mL of wine was initially concentrated in a rotary evaporator under reduced pressure at 50 °C in a water bath. Then, ethanol was added at a 3:1 ratio, and the mixture was kept at 4 °C for 18 h to precipitate the polysaccharides. After precipitation, the samples were centrifuged at 8.000 rpm, dialyzed against distilled water using a membrane with a 6–8 kDa molecular weight cutoff, and lyophilized. To remove residual insoluble compounds, the lyophilized material was solubilized in water (1:10 w/v) and frozen. Successive freezing and thawing cycles were performed until no precipitate was observed after centrifugation.^31^

For the extraction of polysaccharides from Merlot grape pulp, previously lyophilized, seedless, and skinless grapes were ground with water at a 1:5 (w/v) ratio. The mixture was heated to 100 °C, stirred for 8 h, and sonicated for 30 min. It was then centrifuged at 8.000 rpm for 15 min; the supernatant was concentrated, and the polysaccharides were recovered by ethanol precipitation (3:1), followed by dialysis (6–8 kDa) and lyophilization. Freezing, thawing, and centrifugation cycles were performed to remove insoluble materials. The soluble fraction was then concentrated, frozen, and lyophilized.

The proportions of polysaccharides present in the Merlot wine and Merlot grape pulp were determined according to the methodology described by Sassaki et al.^32^

### 2.3 Fractionation of Polysaccharides by Fehling’s Method

Soluble polysaccharide fractions were fractionated using Fehling’s method.^33^ Briefly, they were directly solubilized in an alkaline solution of KOH and sodium potassium tartrate. Then, an equal volume of a 5% CuSO_4_ solution was added under continuous stirring. The mixture was left to stand for 12 h at 4 °C. The resulting precipitate was separated from the supernatant by centrifugation (10,000 rpm, 15 min at 4 °C). Both the precipitate and the supernatant were neutralized with acetic acid and dialyzed. After this procedure, the samples were continuously stirred in an aqueous suspension with cationic resin to remove residual copper and were dialyzed again. The obtained fractions (Fehling’s supernatant and precipitate) were then concentrated and lyophilized.

### 2.4 Homogeneity Test and Molecular Mass Determination

Homogeneity tests were performed using equipment from Wyatt Technology, equipped with a high-performance size exclusion chromatograph (HPSEC). This system included four gel permeation columns in series, with exclusion limits of 7 × 10^6^, 4 × 10^5^, 8 × 10^4^, and 5 × 10^3^ Da, a Waters 2410 refractive index detector, and a multiangle laser light scattering (MALLS) detector at 632.8 nm (Dawn DSP), which measures light scattering at different intensities and angles. A 0.1 mol/L NaNO_3_ solution containing 0.2 g/L NaN_3_ was used as the eluent, with a flow rate of 0.6 mL/min. The samples were dissolved in the eluent solution to a final concentration of 1 mg/mL and filtered through a 0.22 μm cellulose acetate membrane (Millipore). A 200 μL aliquot of each sample was injected while maintaining the eluent flow at 0.6 mL/min. To estimate and calculate the molar mass, the retention time (*R*_T_) of the samples was compared with the *R*_T_ of dextran standards of known molecular masses. The molecular mass was estimated using a calibration curve constructed by linear regression. The chromatograms were analyzed using the ASTRA 4.70.07 software (Wyatt Technology).

### 2.5 Determination of Monosaccharide Composition

Monosaccharide compositions were determined after hydrolyzing 2 mg of polysaccharides with 1 mL of 1 mol/L TFA at 100 °C for 14 h. The solutions were evaporated, and the residue was dissolved in distilled water, followed by the addition of NaBH_4_. After 18 h, the solution was neutralized, dried, and subjected to repeated evaporations with methanol.^34^ The resulting alditols were acetylated with acetic anhydride-pyridine (1:1 v/v) at room temperature overnight. The alditol acetates were extracted with chloroform, and the organic phase was washed several times with a 5% aqueous CuSO_4_ solution until the color of the organic phase matched the initial color of the CuSO_4_ solution. The chloroform phase was then evaporated, and the obtained material was dissolved in acetone for analysis using a Shimadzu gas chromatograph (GC-2010 Plus), coupled to a Shimadzu mass spectrometer (GC-MS-TQ8040). The system operated with a mass scan range of 30–600 *m*/*z* and an electron ionization energy of 70 eV, equipped with an SH-Rtx-5MS capillary column (Shimadzu) coated with fused silica (30 m × 0.25 mm i.d.). Sample injections were performed using the Auto Sampler AOC-5000 Plus system (Shimadzu), starting at 50 °C (held for 1 min), followed by a temperature increase of 40 °C/min to 220 °C, which was then maintained constant until the end of the analysis. Ultrapure helium was used as the carrier gas at a flow rate of 1.0 mL/min. Monosaccharides were identified based on their retention times and fragmentation profiles obtained after electron impact ionization.

### 2.6 Quantification of Uronic Acids

Uronic acid quantification was performed according to the method described by Filisetti-Cozzi and Carpita.^35^ A 0.4 mL sample at a concentration of 1 mg/mL was used, to which 40 μL of a 4 mol/L sulfamic acid-potassium sulfamate solution (pH 1.6) was added. Subsequently, 2.4 mL of sodium tetraborate (75 mmol/L in sulfuric acid) was added. The solution was stirred and heated. After cooling, 80 μL of *meta*-hydroxybiphenyl (0.15% w/v in 0.5% w/v NaOH) was added, and the mixture was stirred again. The presence of uronic acids was indicated by the development of a pink color, with absorbance measured by spectrophotometry (Epoch, BioTek, USA) at 525 nm. Galacturonic acid was used as the standard.

### 2.7 Methylation of Polysaccharides and Analysis as Partially Methylated Alditol Acetates

Polysaccharides were methylated using the method of Ciucanu and Kerek.^36^ The polysaccharide was solubilized in Me_2_SO, followed by the addition of excess NaOH and CH_3_I, with stirring for 30 min, and then left to stand for 24 h. Methylation was stopped by neutralization in an ice bath. The methylated polysaccharide was extracted with chloroform and washed several times with distilled water. The chloroform phase was evaporated, and the material was hydrolyzed with 2 mol/L TFA for 14 h. The hydrolyzed material was dried, reduced with NaBD_4_, and acetylated.^34^ The partially methylated alditol acetates were analyzed using a Shimadzu GC-2010 Plus gas chromatograph coupled to a Shimadzu GC-MS-TQ8040 mass spectrometer, with a mass scan range of 30–600 *m*/*z* and an electron impact ionization energy of 70 eV. The system was equipped with an SH-Rtx-5MS capillary column (Shimadzu) coated with fused silica (30 m × 0.25 mm i.d.). Sample injections were performed using the Auto Sampler AOC-5000 Plus system (Shimadzu), starting at 50 °C (held for 1 min), followed by a temperature increase of 40 °C/min up to 215 °C, which was then maintained constant until the end of the analysis. Ultrapure helium was used as the carrier gas at a flow rate of 1.0 mL/min. The partially methylated alditol acetates were identified based on their retention times and fragmentation profiles.^37^

### 2.8 Collection and Isolation of Salivary Proteins

Whole human saliva was collected from healthy, nonsmoking volunteers (Ethics Committee: 57294922.1.0000.0102), following the protocol of Soares et al.^38,39^ After collection, saliva was immediately treated with TFA (final concentration of 0.1%) to inhibit protease activity and precipitate high-molecular-weight proteins.^38,39^ The sample was then centrifuged (10 min, 12,000*g*), and the supernatant was dialyzed using a cellulose membrane with a 3.5 kDa exclusion limit. The dialyzed material was frozen and lyophilized to obtain salivary proteins.

For the purification process, an ÄKTA Prime Plus liquid chromatography system (GE Healthcare) coupled with a HiTrap Q HP column was used, operating with the system’s preprogrammed anion exchange method. The sample was initially solubilized in 2 mL of buffer A (20 mmol/L *Tris*–HCl, pH 8.0) and subsequently introduced into the ÄKTA system via manual injection through a syringe directly into the sample loop. During the first 10 min of the run, the column was equilibrated with buffer A, followed by sample injection and washing. The elution process was initiated with the introduction of buffer B (20 mmol/L *Tris*–HCl, 1 mol/L NaCl, pH 8.0), and fractions were collected every minute at a flow rate of 1 mL/min. Starting from the injection of buffer B, 37 fractions were collected, grouped according to the obtained chromatogram, dialyzed, and lyophilized.

Total salivary proteins and purified fractions were analyzed by ^1^H NMR and circular dichroism spectrometry. The circular dichroism (CD) spectra were recorded using a JASCO J-815 spectropolarimeter equipped with a Peltier thermostatic cell holder (Model PTC-348WI). The CD measurements were carried out in the range of 250–163 nm, using a cell with a path length of 0.1 cm and salivary protein solutions at a concentration of 0.5 mg/mL in water, with a data pitch of 0.5 nm, a bandwidth of 2 nm, and a scan speed of 20 nm/min. Each spectrum was obtained by averaging three scans. Additionally, total salivary proteins and purified fractions were analyzed by MALDI-ToF/ToF (Autoflex maX by Bruker) in positive reflective mode, using sinapinic acid as the matrix. The results were processed using Compass flexAnalysis (Bruker) software.

### 2.9 Nuclear Magnetic Resonance Spectroscopy

One-dimensional (1D) and two-dimensional (2D) nuclear magnetic resonance (NMR) experiments were acquired in D_2_O for polysaccharide and protein samples and CD_3_OD for phenolic compounds at 70 °C or 30 °C, respectively on an AVANCE III NMR spectrometer (Bruker, Karlsruhe, Germany) operating at 14.1 T, observing the ^1^H and ^13^C nuclei at 600.13 and 150.61 MHz, respectively. The spectrometer was equipped with a 5 mm inverse detection TCI N_2_ Prodigy probe with a z-gradient. ^1^H and ^13^C chemical shifts were determined after ^1^H assignments through COSY and TOCSY (mixing time of 160 ms) analyses.^40^ One-bond and long-range ^1^H–^13^C correlations from HSQCed (positive phase: CH and CH_3_; negative phase: CH_2_) and HMBC NMR experiments were optimized for average one-bond coupling constant ^1^ *J* (H, C) and long-range coupling constant ^LR^ *J* (H, C) of 145 and 8 Hz, respectively. 2D NMR experiments were recorded with quadrature detection in the indirect dimension and acquired using 8–16 scans per series of 1024 × 256 data points, with no zero filling in F1 (1024) before Fourier transformation. ^1^H and ^13^C NMR chemical shifts were given in ppm related to the 3-(trimethylsilyl)­propionic-2,2,3,3*-d*_4_ acid sodium salt (TMSP-*d*_4_) signal at 0.00 ppm as the internal reference.

### 2.10 Saturation Transfer Difference NMR

Saturation transfer difference (STD) NMR experiments were conducted using the water suppression pulse sequence from the Bruker library (stdiffesgp.3). The acquisition conditions for STD NMR spectra used in epitope mapping and titration experiments were as follows: the on-resonance excitation frequency was set at 2289.55 Hz (3.81 ppm), with a spin-lock duration of 25 ms. Off-resonance irradiation was performed at 68440 Hz (114 ppm), outside the spectral window, ensuring that neither polysaccharide nor ligand (phenolic compounds or salivary proteins) signals were present. Selective saturation of polysaccharide hydrogens was achieved using a sequence of Gaussian pulses, each lasting 50 ms and separated by a 1 ms interval. The relaxation time (*d*1) was 4 s, the number of transients (NS) was 256, and the spectral window was 9000 Hz (15 ppm). In titration experiments, the saturation time (*d*20) was varied at 0.5, 0.75, 1.0, 2.0, and 3.0 s for each [L]/[P] ratio. All STD NMR spectra were acquired at 37 °C, and all samples were solubilized in D_2_O.

### 2.11 Determination of the Dissociation Constant (*K*_D_)

Polysaccharide concentration was 5 μM for catechin (Sigma-Aldrich, 43412) titration (100, 150, 200, 250, and 500 μmol/L). STD NMR experiments for *K*_D_ determination were also performed in the presence of 5 μmol/L polysaccharide, catechin, and 3 μmol/L salivary protein to evaluate the effect of protein interference on *K*_D_. Ligands were titrated into polysaccharide samples from concentrated stock solutions to minimize dilution effects. In STD NMR titration experiments, at a given ligand concentration, the STD intensity (ηSTD) of a specific proton is directly proportional to the fraction of bound ligand. The STD amplification factor (STD-AF),^41^ calculated as the product of ηSTD and ligand excess, makes ηSTD dependent on the fraction of bound protein. Thus, plotting STD-AF values against increasing [*L*] produces a dose–response curve.^42^ The binding isotherms were constructed from initial slopes of STD amplification factors (STD-AF_0_), calculated at each ligand concentration throughout the titration.^43^ Each STD-AF_0_ value was obtained by fitting the evolution of STD-AF with saturation time.^44^ The resulting isotherm of initial slopes was mathematically fitted to a Langmuir equation to determine the dissociation constant (*K*_D_). Due to the polydisperse nature of the extracted polysaccharides, the exact number of binding sites (*n*) is unknown. Therefore, the Langmuir model was applied assuming independent and equivalent binding sites. Consequently, the derived values are treated as apparent dissociation constants, providing a robust relative metric for comparison. According to Ângulo et al.,^43^ this method allows for accurate *K*_D_ determination. Additionally, STD-AF_0_ allows epitope mapping by referencing all STD signal intensities to the most intense response, which was normalized to 100%.

## 3 Results and Discussion

### 3.1 Extraction, Purification, Chemical, and Structural Analyses of Polysaccharides

A total of 750 mL of Merlot wine (ME) was used for polysaccharide recovery via ethanol precipitation. After centrifugation, the precipitate was dialyzed, frozen, and lyophilized. The yield, expressed as a percentage relative to the total volume of a standard bottle (750 mL), was 0.110%. The dry material was solubilized in water and subjected to successive freezing and thawing steps to remove any insoluble material. The supernatant obtained after centrifugation (WME) was then frozen and lyophilized, yielding an average recovery of 70% relative to the initial mass.

Additionally, extractions were performed using Merlot grapes obtained from the metropolitan region of Curitiba, PR, Brazil. The aqueous extraction of the pulp (PU) was carried out by heating the previously lyophilized and ground material to 100 °C, followed by agitation, sonication, filtration, and centrifugation. Polysaccharides were recovered via ethanol precipitation, followed by dialysis, freezing, and lyophilization. The polysaccharide yield was approximately 1%, as the pulp is primarily composed of water. The obtained product underwent successive freezing and thawing, followed by centrifugation to remove insoluble materials, and was then lyophilized (WPU). This step aimed to standardize the polysaccharides obtained from the grape with those present in solution in the wine. The yield for the pulp in this procedure was 60%.

The recovered soluble fractions, WME and WPU, were analyzed by high-performance size-exclusion chromatography (HPSEC) (Figure S1). The elution profile was heterogeneous for both fractions, indicating the predominant presence of at least two distinct molecules in the polysaccharide fractions. The estimated molecular masses for the polysaccharides present in the WME fraction were 80.5 and 14.5 kDa, whereas in the WPU fraction, they were 458 and 146 kDa. This difference is primarily due to the winemaking process, which through its various stages can cleave the larger polysaccharides extracted from the grape into smaller fractions present in the wine.

To isolate and/or fractionate the crude soluble sample from the wine (WME), Fehling’s precipitation was employed. This procedure utilizes two solutions: an alkaline solution and a copper­(II) sulfate solution. In an alkaline medium, metal ions form complexes with reactive groups (COOH, OH, NH_2_), leading to the precipitation of molecules. This enables the isolation of specific compounds, as some remain in Fehling’s supernatant while others precipitate. After the procedure, the yield of the supernatant fraction (FSWME) was 71.5%, while the precipitated fraction (FPWME) accounted for 28.5%. Both fractions were analyzed by HPSEC (Figure S1). The FPWME fraction was homogeneous, displaying a single peak with an estimated molecular mass of 74 kDa. In contrast, the FSWME fraction was heterogeneous, with a major peak corresponding to an estimated molecular mass of 11 kDa.

The monosaccharide composition of the WME, FPWME, and FSWME fractions from Merlot wine, as well as the soluble pulp fraction (WPU), was determined (Table 1). The WME fraction exhibited a higher proportion of mannose (∼33%) and galactose (∼25%), followed by galacturonic acid (∼18%), arabinose (∼10%), rhamnose (7%), and glucose (∼7%). After Fehling’s treatment, the FSWME fraction showed a drastic reduction in mannose content, dropping to approximately 3%, with a predominance of galactose (∼42%) and arabinose (33%). Conversely, the FPWME fraction consisted of approximately 95% mannose. Finally, the pulp-derived fraction (WPU) had more than half of its composition as galacturonic acid (54%), along with a high proportion of arabinose (∼28%) and galactose (∼12%). Fehling precipitation was not applied to the WPU fraction, as it did not contain mannose in its composition. As observed for the WME fraction, mannose was the most abundant monosaccharide in the precipitated fraction (FPWME), which explains the affinity of Fehling’s reagent for structures rich in this sugar, due to the presence of vicinal hydroxyl groups in its structure.

**Table 1:** Monosaccharide Composition of the Crude Fractions (WME and WPU) and Fehling’s Fractions (FSWME and FPWME) from Wine and Grape^b^

fraction	monosaccharides (%)					
	Rha	Ara	Man	Glc	Gal	GalA^a^
WME	7.00	10.41	32.68	7.21	24.79	17.92
FSWME	3.16	32.99	3.17	3.71	41.84	15.13
FPWME	-	TR	94.66	-	4.60	-
WPU	3.27	28.58	TR	1.82	11.51	54.09

a GalA was determined using the Filisetti–Cozzi and Carpita^35^ method.

b TR: traces (less than 1%); WME: water-soluble polysaccharide fraction from Merlot wine; FSWME: Fehling supernatant fraction derived from WME; FPWME: Fehling precipitate fraction derived from WME; WPU: water-soluble polysaccharide fraction obtained from grape pulp; Rha: rhamnose; Ara: arabinose; Man: mannose; Glc: glucose; Gal: galactose; GalA: galacturonic acid.

The FPWME fraction contained more than 95% mannose. Mannan is a polysaccharide rich in vicinal hydroxyl groups, which are highly reactive with copper ions, leading to the formation of complexes and the subsequent precipitation of these molecules. This explains the high percentage of mannose in the Fehling precipitate. All fractions were also analyzed by ^1^H and ^13^C­{^1^H} NMR spectra (WME, Figure S2) or HSQCed (FSWME, Figure S3 and Table S1). The presence of the mannan molecule in the FPWME fraction was confirmed by the HSQCed spectrum (Figure 1), as evidenced by signals at 99.1/5.09 (C1/H1), 79.5/4.01 (C2/H2), and 66.5/4.00 ppm (C6/H6), which are characteristic of →2,6)-α-D-Man*p*-(1→ additionally, signals at 101.4/5.27 (C1/H1), 79.3/4.10 (C2/H2), 67.6/3.70 (C4/H4), 74.2/3.76 (C5/H5), and 61.9/3.88 ppm (C6/H6) indicate the presence of →2)-α-D-Man*p*-(1→ signals at 100.5/4.88 (C1/H1), 78.8/3.93 (C2/H2), and 71.4/3.81 ppm (C3/H3) suggest the presence of →6)-α-D-Man*p*-(1→ units. Moreover, signals at 103.1/5.03 (C1/H1), 70.9/4.05 (C2/H2), and 103.0/5.12 (C1/H1), 70.5/4.21 (C2/H2), and 61.9/3.74 ppm (C6/H6) are characteristic of nonreducing terminals α-D-Man*p*-(1→.^45^ These findings suggest the presence of a mannan composed of a main chain of α-D-Man*p* linked (1 → 6) and substituted at *O*-2 by α-D-Man*p-*(1 → 2) residues or chains. Additionally, signals at 104.3/4.45 (C1/H1) and 110.2/5.22 ppm (C1/H1), according to Willför et al.,^46^ indicate the presence of →6)-β-D-Gal*p*-(1→ and α-L-Ara*f*-(1→ derivatives, suggesting possible type II arabinogalactan residues in this fraction.

**Figure 1:**
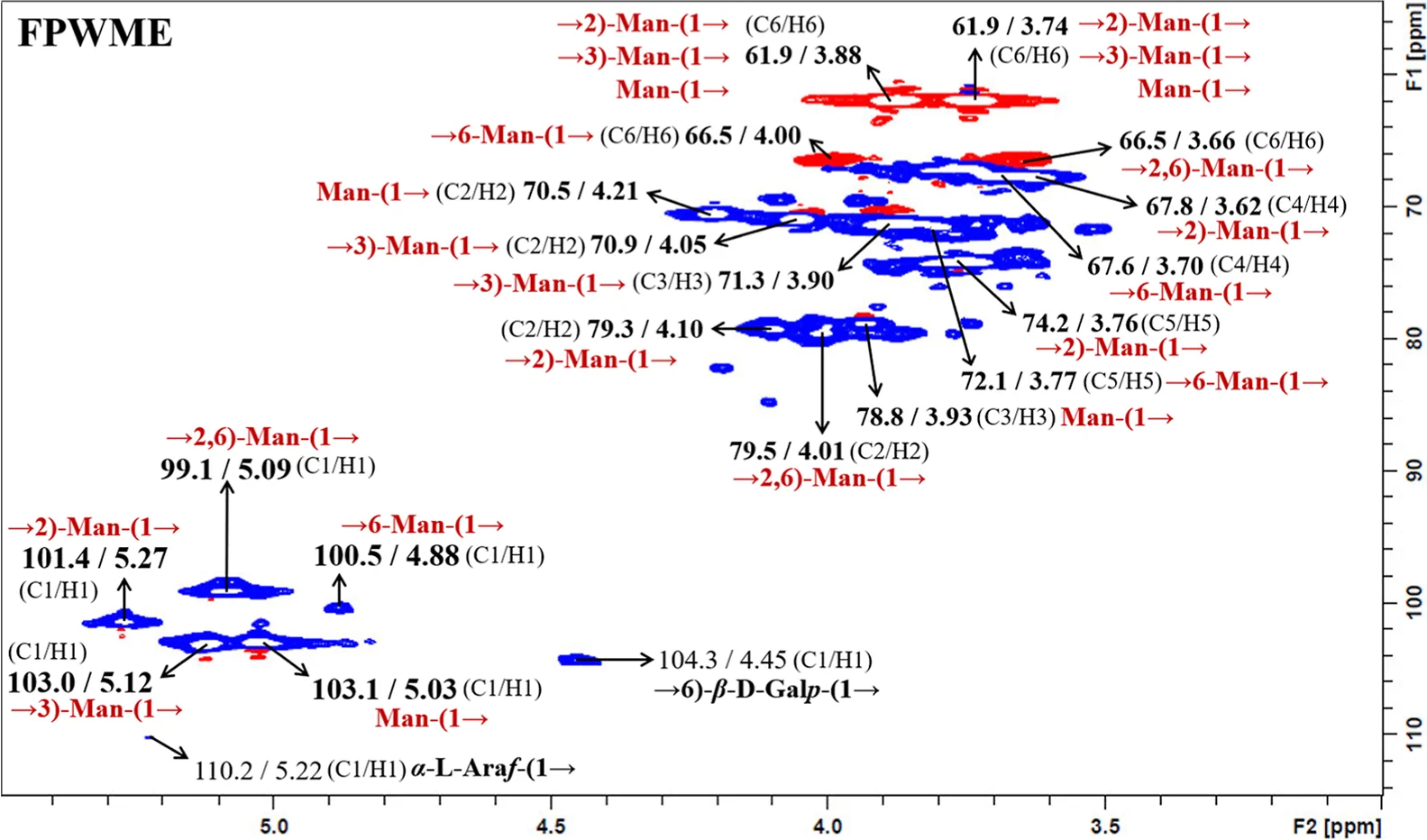
One-bond ^1^H–^13^C correlation map from the HSQCed NMR experiment of the FPWME.

Furthermore, the methylation analysis of the FPWME fraction (Table S2) corroborated the monosaccharide composition and the ^1^H–^13^C HSQC NMR analysis. The results confirmed the presence of a branched polysaccharide, as indicated by the considerable presence of nonreducing terminal units 2,3,4,6-Me_4_-Man. The mannose units substituted at 6-*O* (2,3,4-Me_3_-Man) and 2,6-di-*O* (3,4-Me_2_-Man) suggest a main chain linked (1 → 6), with branching points at the 2-*O* position. The presence of the 3,4,6-Me_3_-Man derivative indicates that these branches consist of mannose chains linked (1 → 2).^47^

The ^1^H–^13^C HSQC NMR analysis of the soluble pulp fraction (WPU) (Figure 2) revealed anomeric chemical shifts (C1/H1) at 104.2/4.46, 103.9/4.50, and 104.4/4.69 ppm, corresponding to the →6)-β-D-Gal*p*-(1→, →3,6)-β-D-Gal*p*-(1→, and →4)-β-D-Gal*p*-(1→ units, respectively. Additional signals at 82.5/3.85 (C3/H3) and 70.0/3.91 ppm (C6/H6) corroborate the presence of →3,6)-β-D-Gal*p*-(1→ residues, while the signal at 83.1/4.19 ppm (C4/H4) confirms →4)-β-D-Gal*p*-(1→ units, and 70.0/4.03 ppm (C6/H6) confirms →6)-β-D-Gal*p*-(1→ unit. The signals at 109.2/5.40 and 109.9/5.24 ppm (C1/H1) are characteristic of nonreducing terminal α-L-Ara*f*. These findings suggest the presence of type I arabinogalactan (AG), which consists of a β-D-Gal*p* (1 → 4)-linked main chain substituted by α-L-Ara*f* units, and type II AG, which features a β-D-Gal*p* main chain linked (1 → 3) and/or (1 → 6), with Gal or Ara branches at 3-*O* and/or 6-*O*.^48–58^

**Figure 2:**
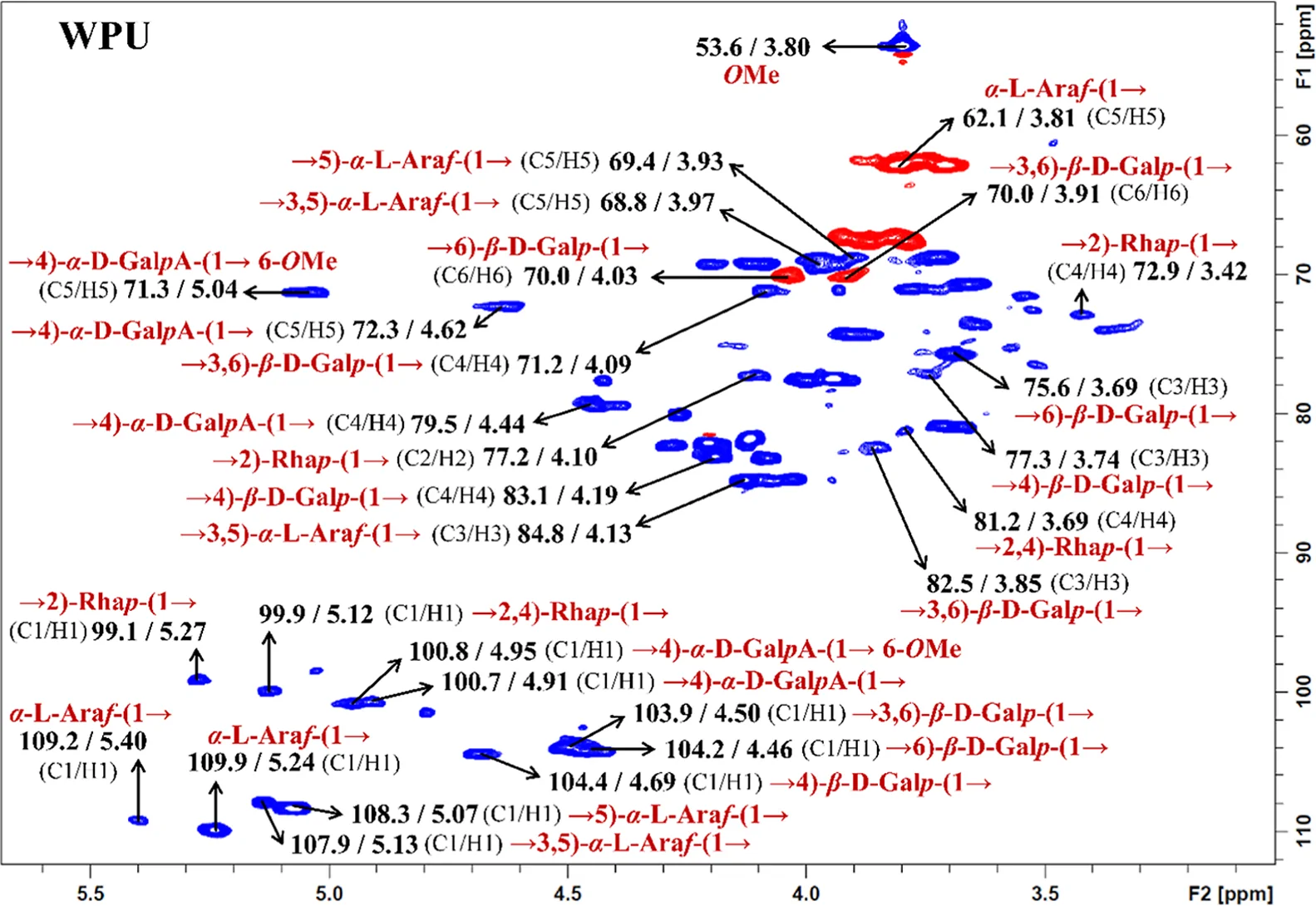
One-bond ^1^H–^13^C correlation map from an HSQCed NMR experiment of the WPU.

The presence of pectins is confirmed by the anomeric signals (C1/H1) at 100.8/4.95 and 100.7/4.91 ppm, corresponding to 6-*O*Me-α-D-Gal*p*A and α-D-Gal*p*A residues, respectively. The shift at 79.5/4.44 ppm (C4/H4) indicates sequences of α-D-Gal*p*A (1→4) linkages, while 71.3/5.04 (C5/H5) and 53.5/3.80 (*O*-Me) corroborate 6-*O*-methyl substitutions.

Methyl-esterified GalA residues are characteristic of type I rhamnogalacturonan (RG). The signals at 99.1/5.27 and 99.9/5.12 ppm (C1/H1) indicate Rha*p* units linked (1 → 2) and (1 → 2,4), further supported by signals at 77.2/4.10 (C2/H2) and 81.2/3.69 ppm (C4/H4). This evidence suggests the presence of type II RG, which consists of α-D-Gal*p*A (1 → 4)-linked residues with *O*-methyl substitutions, and type I RG, composed of α-D-Gal*p*A and Rha*p* units. The presence of arabinan branches is indicated by the signals at 108.3/5.07 and 107.9/5.13 ppm (C1/H1), corresponding to →5)-α-L-Ara*f*-(1→ and →3,5)-α-L-Ara*f*-(1→ units, respectively. The signal at 69.4/3.93 ppm (C5/H5) indicates sequences of α-L-Ara*f* (1 → 5) linkages, while 84.8/4.13 (C3/H3) and 68.8/3.97 (C5/H5) corroborate 3-*O* substitutions. The branching of →2,4)-Rha*p*-(1→ residues suggests that arabinose oligosaccharides are linked to the type I RG main chain via Rha residues.^53,55–60^

The methylation analysis of the WPU fraction (Table S3) confirmed monosaccharide composition and HSQC findings. The results support the presence of AG I, evidenced by the →4)-Gal*p*-(1→ (2,3,6-Me_3_) derivative, which constitutes the main chain. Additionally, the detection of →3,6)-Gal*p*-(1→ (2,4-Me_2_), →3)-Gal*p*-(1→ (2,4,6-Me_3_), and →6)-Gal*p*-(1→ (2,3,4-Me_3_) derivatives confirms the presence of AG II, with Ara*f*-(1→ (2,3,5-Me_3_) terminals residues in both polysaccharides.^48–50,54^ The detection of Rha*p*-(1→ (2,3,4-Me_3_) and →2,4)-Rha*p*-(1→ (3-Me) derivatives suggests the presence of RG I, while the increase in →4)-Gal*p*-(1→ (2,3,6-Me_3_) derivatives after carboxy-reduction confirms the presence of →4)-Gal*p*A-(1→ units.^61^ The →2,4)-Rha*p*-(1→ derivative indicates branched structures, which include →5)-Ara*f*-(1→ (2,3-Me_2_) and →3,5)-Ara*f*-(1→ (2-Me).^62^ Additionally, the presence of the →3,4)-Gal*p*-(1→ derivatives after carboxy-reduction suggests RG structures, characterized by a GalA (1→4)-linked backbone with 3-*O* branching, as proposed by Harholt et al.^62^

Based on the results obtained, the primary monosaccharides identified in the polysaccharide fractions WPU and FPWME were galacturonic acid, arabinose, and galactose in WPU and mannose in FPWME. Galacturonic acid, which constitutes approximately 54% of the WPU fraction, forms sequences of α-D-Gal*p*A (1 → 4)-linked units, indicating the presence of rhamnogalacturonans type I (RG I) and homogalacturonans. Arabinose, accounting for approximately 28%, and galactose, at 12%, are present in arabinogalactans types I and II, contributing to the structural composition of RG I. In the FPWME fraction, mannose represents more than 95% of the total composition, forming a main chain of α-D-Man*p* (1 → 6)-linked units, with branching at *O*-2 by α-D-Man*p* (1 → 2) residues or chains, which is characteristic of mannans.

### 3.2 Obtaining, Purification, and Analyses of Salivary P

Salivary proteins (SPs) were obtained from unstimulated saliva. To facilitate the precipitation of high-molecular-weight SP, reduce viscosity, and preserve sample integrity, trifluoroacetic acid (TFA) was added to a final concentration of 0.1%, which helps to inhibit protease activity. Peptides and proteins such as histatins, proline-rich proteins (PRPs), statherins, cystatins, and defensins remain soluble in the acidic saliva solution and can be recovered after centrifugation.^38,63^ The final yield from saliva collection was 0.04% (0.4 mg/mL) for two distinct donors, corresponding to the lyophilized content of the acidic supernatant. The supernatants were solubilized in D_2_O and analyzed by ^1^H NMR, revealing similar spectral profiles. The absence of a signal at 1.32 ppm in the spectrum (Figure S4) confirmed the efficient removal of residual cellular material from the saliva.^64^ Additionally, intense signals corresponding to proline (Figure S4 and Table S4) confirmed that these supernatant fractions contained the expected protein components.

To identify the proteins present in the acidic supernatant fraction of saliva, MALDI-ToF mass spectrometry was performed in positive reflective mode, using sinapinic acid as the matrix. The obtained spectra are shown in Figure 3. For donor 1, the most intense signals were attributed to peptide P–C (PRPb) (*m*/*z* 4373.076), statherin (Des-Thr_42_-Phe_43_) (PRP) (*m*/*z* 5132.197), statherin (Des-Phe_43_) (PRP) (*m*/*z* 5233.132), peptide P–H (IB-4 - PRPb) (*m*/*z* 5590.800), and II-2 (PRPb) (*m*/*z* 7603.508). Of the 11 identified proteins, eight were PRPs. For donor 2, the most intense signals were attributed to statherin (Des-Phe_43_) (PRP) (*m*/*z* 5233.202), statherin (Des-Thr_42_-Phe_43_) (PRP) (*m*/*z* 5134.597), histatin 2 (*m*/*z* 4841.531), peptide P–B (PRPb) (*m*/*z* 5792.064), peptide P–B (Des_1–12_) (PRPb) (*m*/*z* 4549.299), and peptide P–C (PRPb) (*m*/*z* 4372.188). Among the ten identified proteins, eight were PRPs.^38,63,65–69^

**Figure 3:**
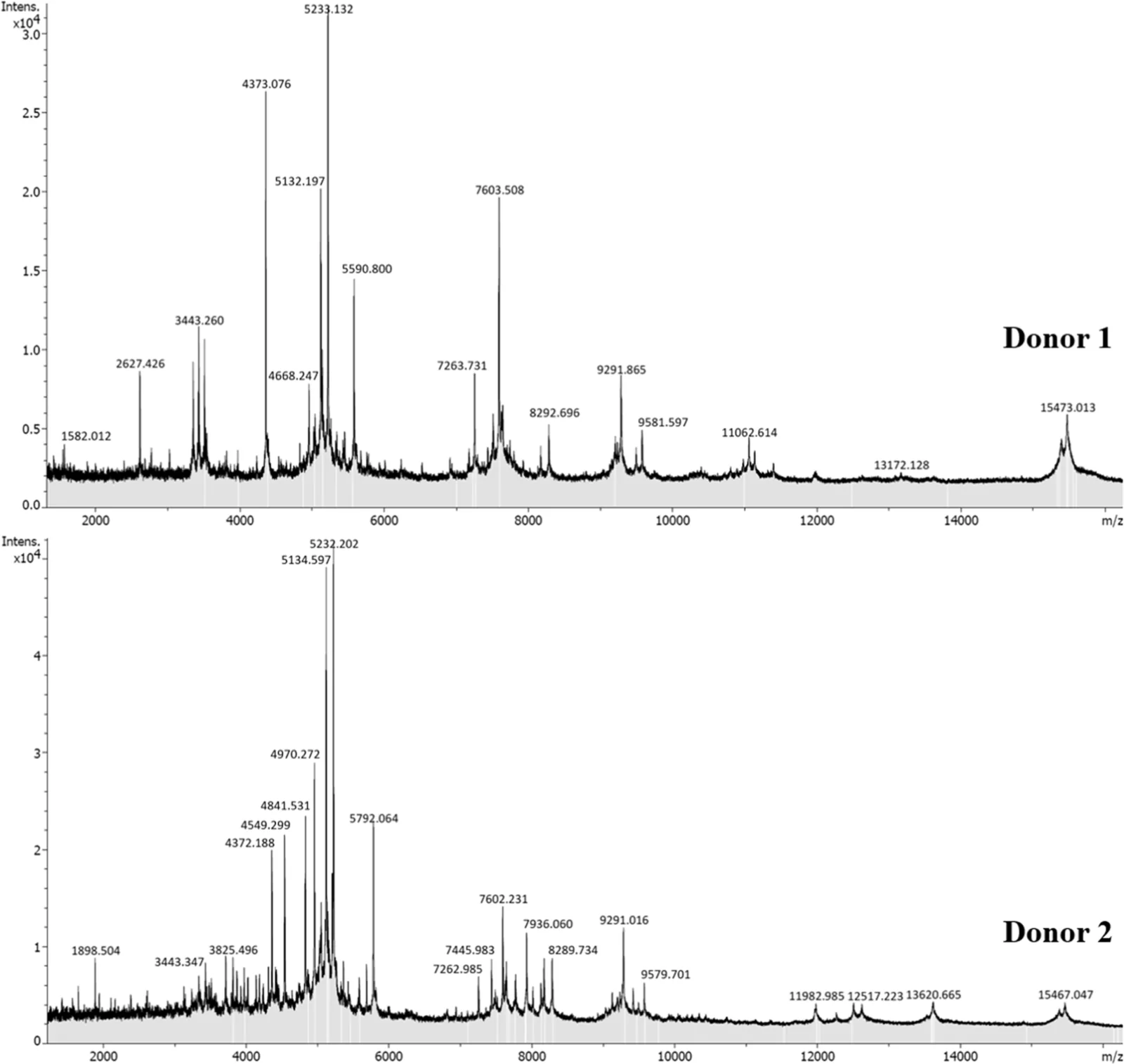
MALDI-ToF spectra of the salivary supernatant in 0.1% TFA.

PRPs were detected in high proportions in both samples. However, due to the presence of α-defensin 1, histatins, and additional metabolites detected by ^1^H NMR, further purification was required. For this purpose, anion exchange chromatography was performed using a HiTrap Q HP column coupled to a liquid chromatography system. For donor 1, 37 fractions were collected (Figure S5). The most intense signals were found in fractions 15, 16, and 17, which were subsequently pooled. A similar procedure was applied for donor 2, where fractions eluted in the first 10 min were collected (Figure S5). The pooled fractions were dialyzed against distilled water at 4 °C for 48 h, using 3.5 kDa exclusion limit membranes, with periodic buffer exchanges to remove residual salts. The dialyzed fractions were then frozen and lyophilized.

The purified fractions were analyzed by MALDI-ToF mass spectrometry. For group 1, composed of fractions 15, 16, and 17, the mass spectrum (Figure 4) exhibited a prominent signal at *m*/*z* 5131.571, corresponding to statherin.^38,66^ Statherin is an acidic peptide rich in proline and tyrosine, with biological functions similar to those of acidic PRPs.^70,71^ For group 2, corresponding to fractions collected within the first 10 min (Figure 4), signals were observed at *m*/*z* 5063.747, 5217.291, 5374.192, and 5794.367, corresponding to peptide P–B (Des_1–3_), peptide P–B (Des_1–5_), peptide P–B (Des_1–7_), and peptide P–B, respectively.^38,66^ These assignments confirm the presence of peptide P–B, a member of the basic PRP family.^71,72^

**Figure 4:**
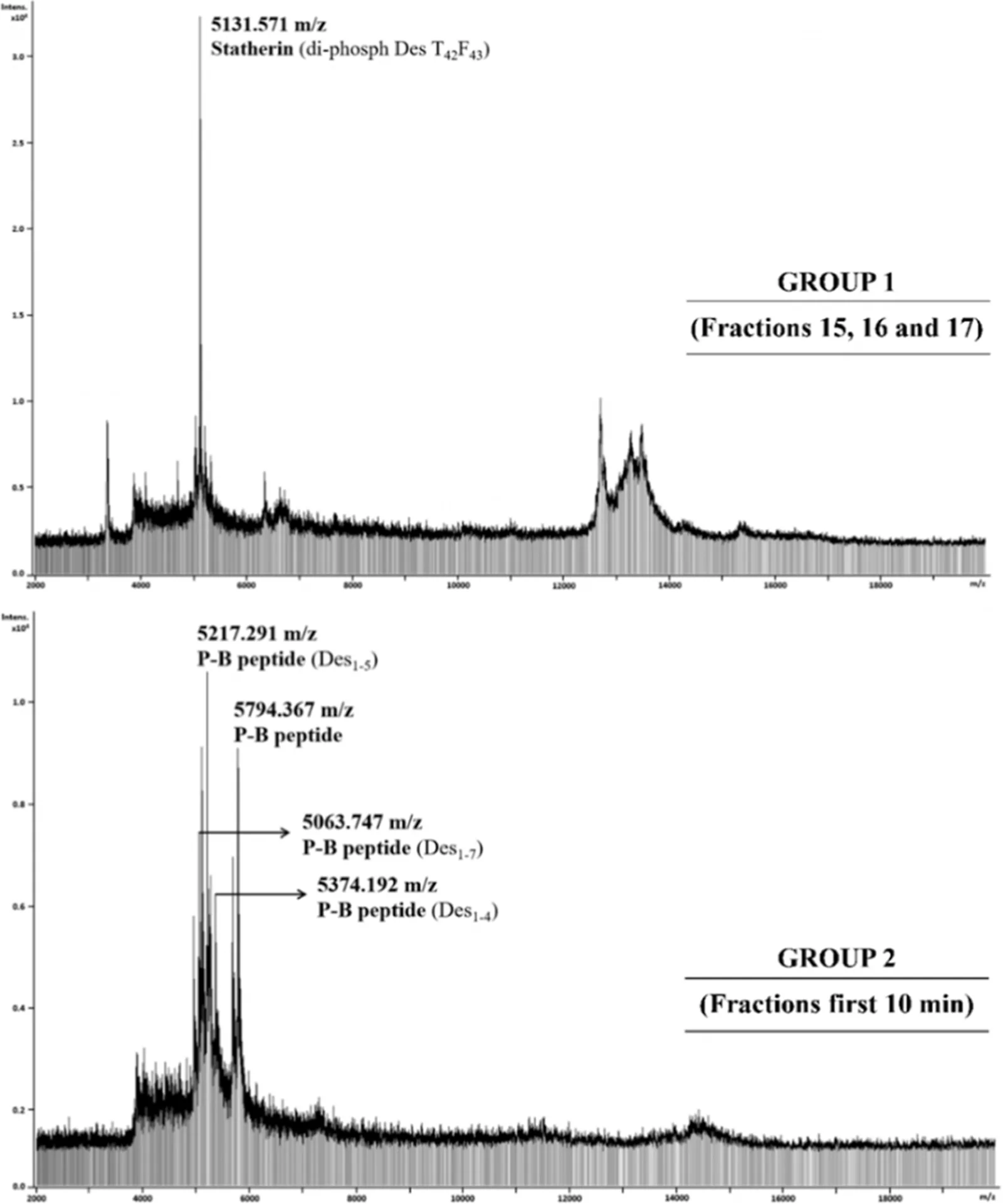
MALDI-ToF spectra of purified fractions.

### 3.3 Interactions between Polysaccharides, Catechins, and PRPs

The perception of astringency in wines is influenced not only by the endogenous phenolic compounds and their interaction with salivary proteins but also by other constituents, such as carbohydrates.^25,73^ Polysaccharides play a significant role in modulating astringency through two main mechanisms: a direct effect and an indirect effect. In the direct mechanism, polysaccharides bind to free phenolic compounds, effectively sequester them, and reduce their bioavailability. This prevents the polyphenols from cross-linking with salivary proteins, thereby reducing the precipitation of the protein–tannin complexes responsible for the loss of oral lubrication. In the indirect mechanism, polysaccharides can interact directly with salivary proteins or form soluble ternary complexes (polysaccharide–polyphenol–protein). This competitive interference either shields the proteins from precipitation or increases the overall solubility of the aggregates, ultimately mitigating the astringent sensation.^74^ Furthermore, evidence suggests that the intensity of astringency is more strongly correlated with the strength of the interaction between salivary proteins and phenolic compounds than with the ability of polyphenols to precipitate these proteins.^19^ Therefore, evaluating the strength of these interactions and the influence of salivary proteins on polysaccharide–phenolic interactions are essential for a deeper understanding of the inherent astringency of wines.

In light of these considerations, interaction experiments were conducted using 3 μmol/L of each previously isolated PRP group,^75^ while catechin (catechin hydrate, Sigma-Aldrich, C1251) was employed at increasing concentrations ranging from 100 to 500 μmol/L. The concentration of polysaccharides was set at 5 μmol/L for each, including a mannan (neutral polysaccharide) extracted from wine and a pectic polysaccharide derived from grape pulp (charged polysaccharide), both solubilized in D_2_O along with the PRPs and catechin. Protons that exhibited signals in the STD spectrum were used to determine the dissociation constant (*K*_D_) and to map the ligand epitope. The STD amplification factor (STD-AF) was calculated based on the differences between the on-resonance and off-resonance spectra as catechin concentration increased. The corresponding STD plots, derived from the initial slope of the STD-AF values (αSTD_0_), were analyzed to characterize the interaction between grape pulp polysaccharides (Figure 5—right) and mannan (Figure 5—left) with catechin, aPRP, and bPRP. These data were then used to obtain the best mathematical fit to the Langmuir isotherm equation (Figure 5—dashed lines), from which the apparent *K*_D_ values were extracted.

**Figure 5:**
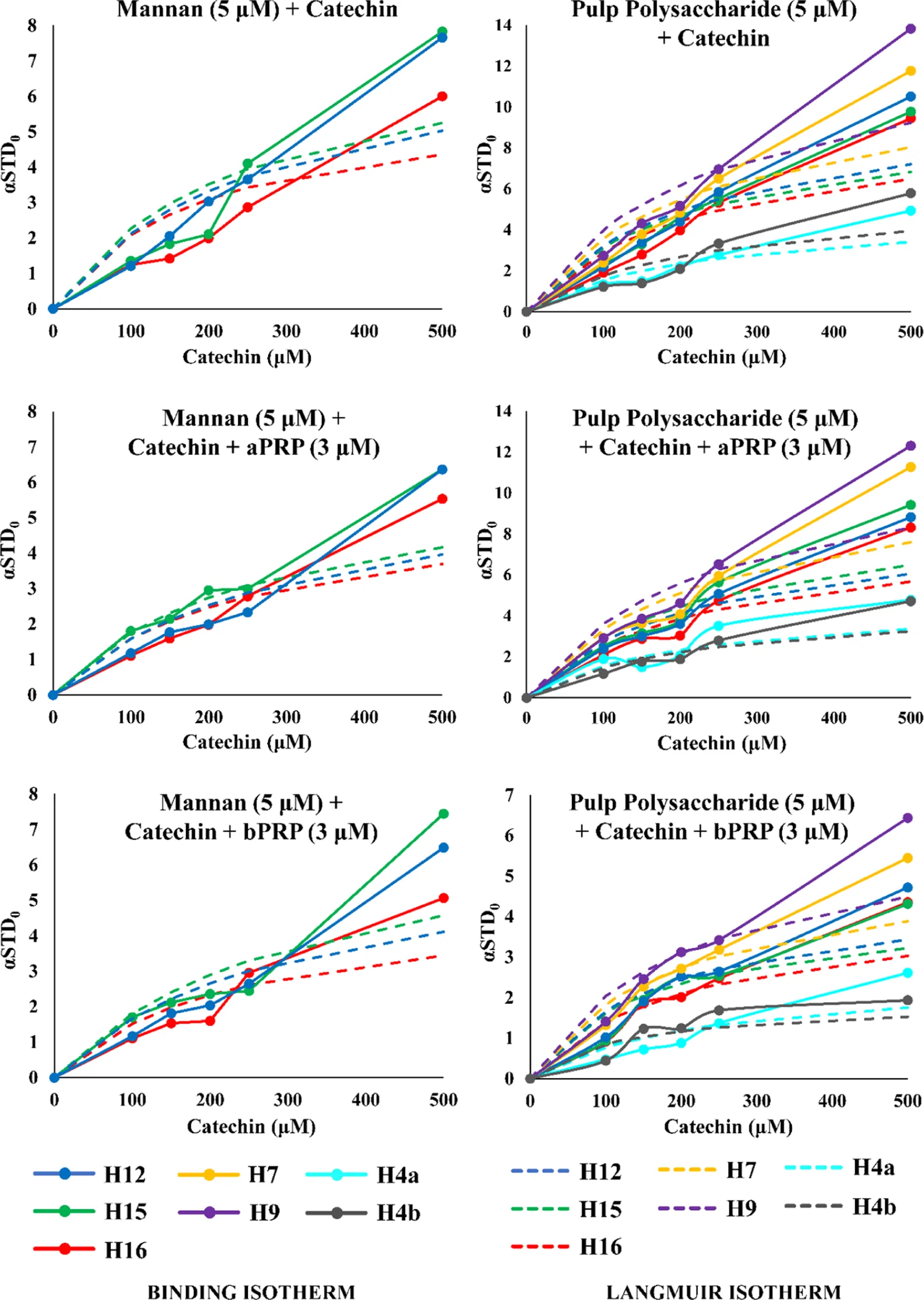
Initial slope (αSTD_0_) of the STD-AF values as a function of ligand concentration and mathematical fit to the Langmuir isotherm (dashed lines) for both polysaccharides interacting with catechin, in the presence or absence of PRPs.

Table 2 presents the *K*_D_ values obtained for all of the studied associations. In the absence of PRPs, the *K*_D_ values for both polysaccharides interacting with catechin are nearly identical. Although previous studies suggest that neutral polysaccharides exhibit weaker association with phenolic compounds compared to those rich in acidic sugars,^76,77^ no significant difference was observed here between the two polysaccharides in terms of catechin binding. Upon the addition of aPRP, no change in the *K*_D_ is detected for the pulp polysaccharide. However, in the case of mannan, the presence of either aPRP or bPRP results in a noticeable increase in the *K*_D_. Given that a higher *K*_D_ value corresponds to a lower binding affinity, these findings suggest that both PRP groups can interfere with the interaction between mannan and catechin.

**Table 2:** Dissociation Constants (*K*_D_) [μM] Determined from the Initial Slopes of STD-AF Growth Rates for Mannose-Ligand Systems, with or without PRPS, Based on the STD Signals of Different Ligand Protons^a^

polysaccharide		mannan			pulp (pectin)		
protein–		aPRP	bPRP	–	aPRP	bPRP	
Position	^1^H (ppm)	*K*_D_ (μM)	*K*_D_ (μM)	*K*_D_ (μM)	*K*_D_ (μM)	*K*_D_ (μM)	*K*_D_ (μM)
4a	2.50	–	–	–	223	211	244
4b	2.85	–	–	–	232	226	133
7	5.92	–	–	–	232	242	201
9	5.85	–	–	–	249	240	216
12	6.83	188	249	234	228	233	218
15	6.76	245	264	314	215	225	168
16	6.71	261	303	289	230	227	187
apparent *K*_D_ (μM)		231 ± 38	272 ± 28	279 ± 41	230 ± 10	229 ± 10	195 ± 37

a The numbers in column H represent hydrogen atoms from the catechin structure (Figure 6) that showed interaction with the respective polysaccharides. Their chemical shifts are presented, as well as the *K*_D_ values for each one and the corresponding average (±standard deviation). aPRP: acidic proline-rich proteins; bPRP: basic proline-rich proteins.

By mapping the catechin epitopes through the identification of the hydrogen atoms involved in the interaction, it is possible to elucidate the chemical characteristics underlying the binding process. As shown in Figure 6, in the case of mannan, it is observed that the interaction occurs exclusively on the aromatic B ring in the presence of both PRPs. This indicates that the binding is primarily governed by a combination of noncovalent forces, notably CH-π interactions (between the apolar patches of the mannan pyranose rings and the π-system of the catechin) and the classic solvent-mediated hydrophobic effect. Furthermore, the addition of a PRP, whether acidic or basic, can effectively influence the binding of catechin to mannan. This competitive effect is in line with previous reports indicating that certain phenolic compounds preferentially and exclusively bind to PRPs through hydrophobic interactions.^75,78^ PRPs exhibit a high affinity for phenolic compounds due to the presence of proline residues, which facilitate the formation of hydrogen bonds and hydrophobic interactions.^79,80^ This phenomenon explains the increase in *K*_D_ values for interactions involving both PRPs.

**Figure 6:**
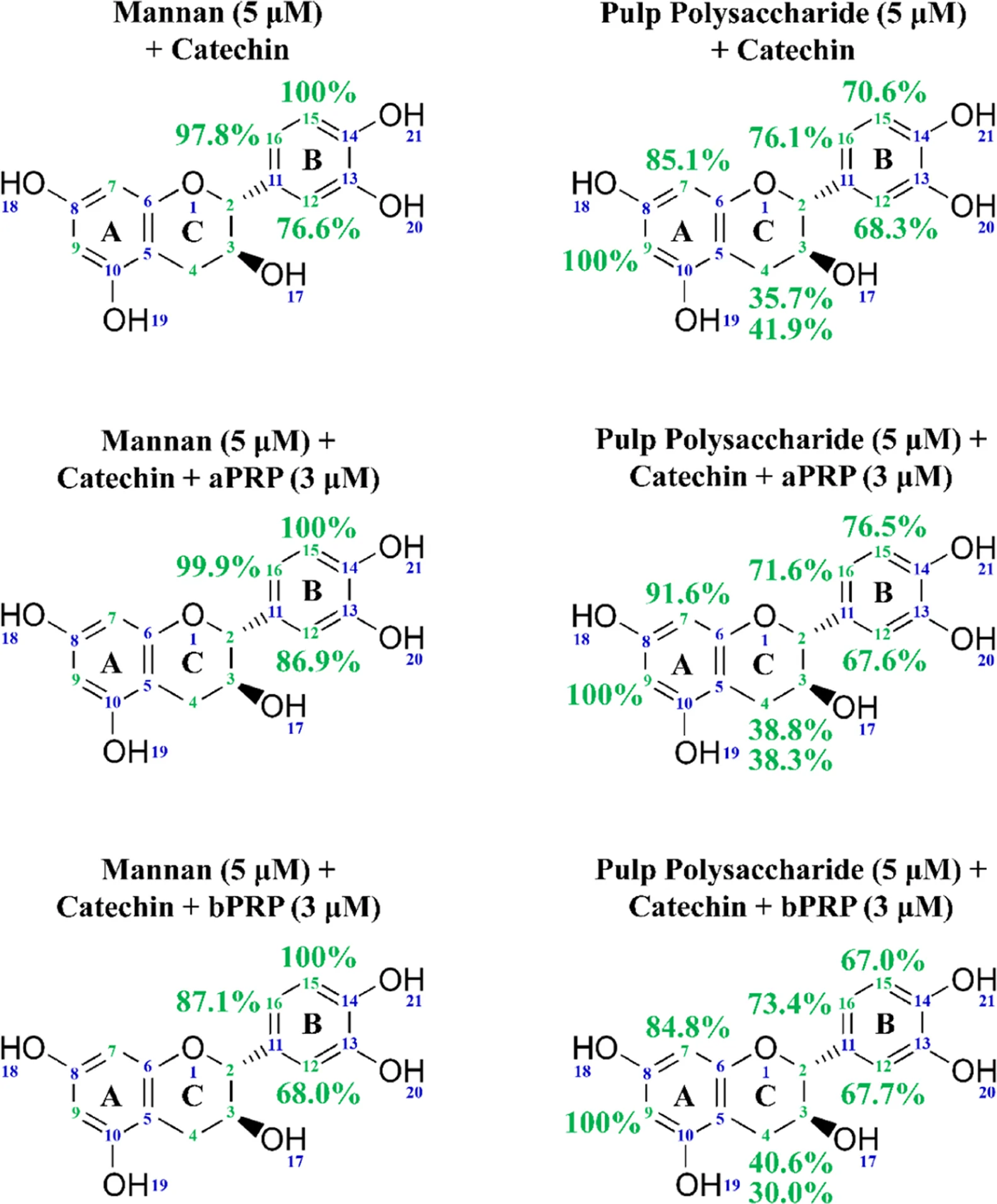
Ligand epitope map for a saturation time limit approaching zero (αSTD_0_) for both polysaccharides, with and without PRP association.

The competitive nature of this interaction is strongly supported by the literature on direct PRP-polyphenol binding. Studies by Jahmidi-Azizi et al. (2023),^81^ Dufourc et al.(2024),^82^ and Tortorella et al.(2026)^83^ have demonstrated that proline-rich proteins and structural models (such as poly-l-proline) exhibit binding affinities (*K*_A_) for catechins typically in the range of 10^3^ to 10^4^ M^–1^ (corresponding to *K*_D_ values in the micromolar range). The baseline apparent *K*_D_ determined in our study for the mannan–catechin interaction (≈231 μM) falls within this same order of magnitude. This thermodynamic parity explains why the addition of PRPs efficiently disrupts the polysaccharide–catechin equilibrium. Because the catechin’s affinity for PRPs is comparable to its affinity for the neutral polysaccharide, the PRPs act as highly effective competitors, displacing the catechin from the mannan and consequently increasing the apparent *K*_D_ of the complex.

When analyzing the interaction involving polysaccharides extracted from grape pulp (Figure 6—right), it is observed that binding occurs at the A, C, and B rings of catechin. This suggests the involvement of other types of interactions beyond hydrophobic forces. Acidic polysaccharides, such as pectins, which are the main components of grape pulp polysaccharides, tend to form bonds with phenolic compounds due to the presence of carboxyl (–COO^–^) groups in their structures. These groups can interact with the hydroxyl groups of phenolic compounds through hydrogen bonding and electrostatic interactions.^84,85^ Furthermore, the high density of negative charge in pectins can facilitate the formation of stable complexes with phenolic compounds.^85^

Thus, the lack of change in *K*_D_ values when aPRP is added to the pectin–catechin interaction can be explained by the fact that this PRP does not compete with or interfere with all types of bonds involved in this interaction, affecting only hydrophobic interactions. Additionally, the negative charges of aPRP do not significantly alter the overall charge of pectin, maintaining its ability to interact with the phenolics. This explains why aPRP does not significantly modify the *K*_D_ value of the interaction with pectin when compared with the *K*_D_ observed for mannan, a neutral polysaccharide.

Conversely, the presence of bPRP results in a decrease in the *K*_D_ value of the pectin–catechin interaction, indicating an increase in binding affinity between these molecules. This effect can be attributed to favorable electrostatic interactions between the opposite charges of the involved molecules. Being basic, bPRP contains amino acid residues with positive charges, which can interact with the negatively charged carboxyl groups of pectin, reducing electrostatic repulsion between pectin’s chains and creating a more favorable environment for catechin binding. Unlike the neutral mannan system, where PRPs act as independent competitive sinks due to their comparable baseline affinity for catechin, bPRP interacts directly with the pectin backbone. Because bPRP is sequestered by the polysaccharide through these strong electrostatic associations, it does not compete directly for the catechin but rather forms a synergistic macromolecular complex that maintains or even enhances pectin’s ability to interact with the phenolic compounds.

Interestingly, in the absence of proteins, the *K*_D_ values for catechin interactions with both polysaccharides (pectin and mannan) are similar, suggesting a comparable baseline binding behavior. This likely occurs because, without protein mediation, noncovalent forces such as CH-π and hydrophobic effects predominantly govern the interaction. However, the introduction of proteins shifts these interaction dynamics, affecting the polysaccharide–catechin complexes in distinct ways depending on their specific structural conformations.

In conclusion, this study demonstrates that wine polysaccharides are not mere passive components, but active modulators of astringency, exhibiting a clear dual and opposing regulatory pattern that is highly dependent on their distinct physicochemical properties. Using STD-NMR, we provided molecular-level evidence that neutral and charged polysaccharides interact differently with catechin in the presence of salivary proteins. For neutral polysaccharides like mannan, the addition of both acidic and basic PRPs creates a competitive environment (antagonistic pattern). The PRPs compete for the hydrophobic and CH-π binding sites, effectively increasing the dissociation constant (*K*_D_) and weakening the mannan–catechin interaction. In contrast, for acidic, negatively charged polysaccharides like pectin, the interaction mechanism is distinct and highly cooperative (synergistic pattern). While acidic PRPs show minimal interference, basic PRPs (bPRP) actively cooperate. The positively charged bPRP neutralizes electrostatic repulsions within the pectin matrix, significantly lowering the *K*_D_ and enhancing the binding affinity between pectin and catechin. Although these thermodynamic shifts are modest, reflecting the dynamic, weak noncovalent equilibria inherent to wine, the epitope mapping confirms fundamental structural changes in the binding complexes. Ultimately, understanding these competitive and cooperative mechanisms provides a deeper molecular basis for how polysaccharides can be strategically employed to refine and stabilize the sensory quality and astringency of wines.

## Supplementary Material



## Notes

Figure S1: elution profiles of soluble polysaccharide fractions determined by HPSEC-RID; Figure S2: 1D ^1^H and ^13^C NMR spectra of the water-soluble polysaccharide fraction from Merlot wine; Figure S3: 2D HSQC NMR spectrum and signal assignments for the Fehling supernatant fraction; Figure S4: ^1^H NMR spectrum (600 MHz) of the acidic supernatant fraction with identified compounds; Figure S5: anion exchange chromatograms of salivary protein samples from two different donors; Table S1: alditol acetate derivatives and glycosidic linkages of the FSWME fraction before and after carboxy-reduction; Table S2: GC–MS fragmentation profile and percentages of alditol acetate derivatives from the FPWME fraction; Table S3: methylation analysis, glycosidic linkages, and monosaccharide composition of the grape pulp fraction; and Table S4: list of amino acids, organic acids, and other metabolites identified in the acidic supernatant fraction by NMR (PDF)

## Abbreviations

AG: arabinogalactan
aPRP: acidic proline-rich proteins
Ara: arabinose
bPRP: basic proline-rich proteins
CD: circular dichroism
COSY: correlation spectroscopy
CuSO_4_: copper­(II) sulfate
D_2_O: deuterium oxide
DMSO (Me_2_SO): dimethyl sulfoxide
FPWME: Fehling precipitate fraction derived from WME
FSWME: Fehling supernatant fraction derived from WME
Gal: galactose
GalA: galacturonic acid
GC-MS: gas chromatograph coupled to a mass spectrometer
Glc: glucose
HMBC: heteronuclear multiple bond correlation
HPLC: high-performance liquid chromatography
HPSEC: high-performance size exclusion chromatography
HSQCed: edited heteronuclear single quantum coherence
*K*_D_: dissociation constant
KOH: potassium hydroxide
LR_*J*_: long-range coupling constant
MALDI-ToF: matrix-assisted laser desorption/ionization time-of-flight
MALLS: multiangle laser light scattering
Man: mannose
NaBD_4_: sodium borodeuteride
NaBH_4_: sodium borohydride
NaN_3_: sodium azide
NaNO_3_: sodium nitrate
NMR: nuclear magnetic resonance
PRP: proline-rich salivary proteins
RG-I: rhamnogalacturonans type I
RG-II: rhamnogalacturonans type II
Rha: rhamnose
*R*_T_: retention time
SP: salivary proteins
STD NMR: saturation transfer difference nuclear magnetic resonance
STD-AF: STD amplification factor
STD-AF_0_: initial slopes of STD amplification factors
TFA: trifluoroacetic acid
TMSP-*d*_4_: 3-(trimethylsilyl)­propionic-2,2,3,3-d4 acid sodium salt
TOCSY: total correlation spectroscopy
WME: water-soluble polysaccharide fraction from Merlot wine
WPU: water-soluble polysaccharide fraction obtained from grape pulp

## References

[1] Lingua M. S., Fabani M. P., Wunderlin D. A., Baroni M. V. (2016). From grape to wine:
Changes in phenolic composition and its influence on antioxidant activity. Food Chem..

[2] Gutiérrez-Escobar R., Aliaño-González M. J., Cantos-Villar E. (2021). Wine polyphenol
content and its influence on wine quality and properties: A review. Molecules.

[3] Guerra, C. C. ; Mandelli, F. ; Tonietto, J. ; Zanus, M. C. ; Camargo, U. A. Conhecendo o essencial sobre uva e vinhos. In Bento GonçAlves, RS; Embrapa Uva e Vinho (Documentos, 2005; Vol. 48.

[4] Waterhouse A. L. (2002). Wine phenolics. Ann. N.Y. Acad. Sci..

[5] Sirén H., Sirén K., Sirén J. (2015). Evaluation of organic and inorganic
compounds levels of red wines processed from Pinot Noir grapes. Anal. Chem. Res..

[6] Martínez-Lapuente, L. , Guadalupe, Z. , Ayestarán, B. (2019). Properties of Wine Polysaccharides. In: Masuelli, M. , Ed., Pectins - Extraction, Purification, Characterization and Applications; IntechOpen. DOI: 10.5772/intechopen.85629.

[7] Vidal S., Williams P., Doco T., Moutounet M., Pellerin P. (2003). The polysaccharides of red wine: Total fractionation
and characterisation. Carbohydr. Polym..

[8] Waters E. J., Pellerin P., Brillouet J. M. (1994). A saccharomyces
mannoprotein that
protects wine from protein haze. Carbohydr.
Polym..

[9] Nemzer B., Kalita D., Yashin A. Y., Yashin Y. I. (2022). Chemical Composition
and Polyphenolic Compounds of Red Wines: Their Antioxidant Activities
and Effects on Human Health A Review. Beverages.

[10] Lunte S. M., Blankenship K. D., Read S. A. (1988). Detection and identification
of procyanidins
and flavanols in wine by dual-electrode liquid chromatography-electrochemistry. Analyst.

[11] Decendit A., Waffo-Teguo P., Richard T., Krisa S., Vercauteren J., Monti J. P., Deffieux G., Mérillon J. M. (2002). Galloylated
catechins and stilbene diglucosides in *Vitis vinifera* cell suspensions cultures. Phytochemistry.

[12] Mattivi F., Guzzon R., Vrhovsek U., Stefanini M., Velasco R. (2006). Metabolite profiling of grape: Flavonols
and anthocyanins. J. Agric. Food Chem..

[13] Gawel R. (1998). Red wine astringency:
a review. Aust. J. Grape Wine Res..

[14] Manconi B., Castagnola M., Cabras T., Olianas A., Vitali A., Desiderio C., Sanna M. T., Messana I. (2016). The intriguing heterogeneity
of human salivary proline-rich proteins. J.
Proteom..

[15] Bennick A. (2002). Interaction
of Plant Polyphenols with Salivary Proteins. Crit. Rev. Oral Biol. Med..

[16] Lu Y., Bennick A. (1998). Interaction of tannin
with human salivary proline-rich
proteins. Arch. Oral Biol..

[17] Quijada-Morín N., Crespo-Expósito C., Rivas-Gonzalo J. C., García-Estévez I., Escribano-Bailón M. T. (2016). Effect
of the addition of flavan-3-ols on the HPLC-DAD salivary-protein profile. Food Chem..

[18] Brandão E., Soares S., Mateus N., De Freitas V. (2014). In vivo interactions
between procyanidins and human saliva proteins: effect of repeated
exposures to procyanidins solution. J. Agric.
Food Chem..

[19] Obreque-Slier E., Lopez-Solis R., Pena-Neira A., Zamora-Marin F. (2010). Tannin-protein
interaction is more closely associated with astringency than tannin-protein
precipitation: experience with two oenological tannins and a gelatin. Int. J. Food Sci. Technol..

[20] Guadalupe, Z. , Ayestaran, B. , Williams, P. , Doco, T. (2014). Determination of must and wine polysaccharides by gas chromatography-mass spectrometry (GC-MS) and size-exclusion chromatography (SEC). In: Ramawat, K. , J. M., Mérillon , Eds., Polysaccharides. Springer, Cham. DOI: 10.1007/978-3-319-03751-6_56-2.

[21] Riou V., Vernhet A., Doco T., Moutounet M. (2002). Aggregation
of grape seed tannins in model wine-effect of wine polysaccharides. Food Hydrocolloids.

[22] Watrelot A. A., Le Bourvellec C., Imberty A., Renard C. M. G. C. (2013). Interactions
between pectic compounds and procyanidins are influenced by methylation
degree and chain length. Biomacromolecules.

[23] Watrelot A. A., Le Bourvellec C., Imberty A., Renard C. M. G. C. (2014). Neutral sugar
side chains of pectins limit interactions with procyanidins. Carbohydr. Polym..

[24] Brandão E., Silva M. S., García-Estévez I., Williams P., Mateus N., Doco T., De Freitas V., Soares S. (2020). Inhibition mechanisms of wine polysaccharides on salivary
protein precipitation. J. Agric. Food Chem..

[25] Boulet J. C., Trarieux C., Souquet J. M., Ducasse M. A., Caillé S., Samson A., Williams P., Doco T., Cheynier V. (2016). Models based
on ultraviolet spectroscopy, polyphenols, oligosaccharides and polysaccharides
for prediction of wine astringency. Food Chem..

[26] Hanlin R. L., Hrmova M., Harbertson J. F., Downey M. O. (2010). Review: Condensed
tannin and grape cell wall interactions and their impact on tannin
extractability into wine. Aust. J. Grape Wine
Res..

[27] Jones-Moore H. R., Jelley R. E., Marangon M., Fedrizzi B. (2022). The interactions of
wine polysaccharides with aroma compounds, tannins, and proteins,
and their importance to winemaking. Food Hydrocolloids.

[28] Takeuchi K., Wagner G. (2006). NMR studies of protein
interactions. Curr. Opin. Struct. Biol..

[29] Furukawa A., Konuma T., Yanaka S., Sugase K. (2016). Quantitative analysis
of protein–ligand interactions by NMR. Prog. Nucl. Magn. Reson. Spectrosc..

[30] Dalvit C., Gmür I., Rößler P., Gossert A. D. (2023). Affinity measurement
of strong ligands with NMR spectroscopy: Limitations and ways to overcome
them. Prog. Nucl. Magn. Reson. Spectrosc..

[31] Gorin P. A. J., Iacomini M. (1984). Polysaccharides of
the lichens *Cetraria islandica* and *Ramalina
usnea*. Carbohydr.
Res..

[32] Sassaki G. L., Guerrini M., Serrato R. V., Santana Filho A. P., Carlotto J., Simas-Tosin F., Cipriani T. R., Iacomini M., Torri G., Gorin P. A. J. (2014). Monosaccharide
composition of glycans
based on Q-HSQC NMR. Carbohydr. Polym..

[33] Jones, J. K. N. , Stoodley, R. J. (1965). Fractionation Using Copper Complexes. In: Whistler, R. L. , Ed., Methods in Carbohydrate Chemistry; Academic Press, 5, 36–38.

[34] Wolfrom M. L., Thompson A. (1963). Reduction with sodium borohydride. Methods Carbohydr. Chem..

[35] Filisetti-Cozzi T. M., Carpita N. C. (1991). Measurement of uronic acids without interference from
neutral sugars. Anal. Biochem..

[36] Ciucanu I., Kerek F. (1984). A simple and rapid
method for the permethylation of carbohydrates. Carbohydr. Res..

[37] Sassaki G. L., Gorin P. A. J., Souza L. M., Czelusniak P. A., Iacomini M. (2005). Rapid synthesis of partially *O*-methylated
alditol acetate standards for GC–MS: some relative activities
of hydroxyl groups of methyl glycopyranosides on Purdie methylation. Carbohydr. Res..

[38] Messana I., Cabras T., Inzitari R., Lupi A., Zuppi C., Olmi C., Fadda M. B., Cordaro M., Giardina B., Castagnola M. (2004). Characterization of the human salivary
basic prolinerich
protein complex by a proteomic approach. J.
Proteome Res..

[39] Soares S., Vitorino R., Osório H., Fernandes A., Venâncio A., Mateus N., Amado F., De Freitas V. (2011). Reactivity
of human salivary proteins families toward food polyphenols. J. Agric. Food Chem..

[40] Galinari E., Sabry D. A., Sassaki G. L., Macedo G. R., Passos F. M. L., Mantovani H. C., Rocha H. A. O. (2017). Chemical structure, antiproliferative
and antioxidant activities of a cell wall α-D-mannan from yeast *Kluyveromyces marxianus*. Carbohydr.
Polym..

[41] Mayer M., Meyer B. (2001). Group epitope mapping
by saturation transfer difference NMR to identify
segments of a ligand in direct contact with a protein receptor. J. Am. Chem. Soc..

[42] Lepre C. A., Moore J. M., Peng J. W. (2004). Theory and applications of NMR-based
screening in pharmaceutical research. Chem.
Rev..

[43] Ângulo J., Enríquez-Navas P. M., Nieto P. M. (2010). Ligand-receptor
binding affinities from saturation transfer difference (STD) NMR spectroscopy:
the binding isotherm of STD initial growth rates. Chemistry.

[44] Mayer M., James T. L. (2004). NMR-Based Characterization of Phenothiazines as a RNA
Binding Scaffold. J. Am. Chem. Soc..

[45] Kobayashi H., Watanabe M., Komido M., Matsuda K., Ikeda-Hasebe K., Suzuki M., Shibata N., Hisamichi K., Suzuki S. (1995). Assignment of ^1^H and ^13^C NMR
chemical shifts of a D-mannan composed of α-(1→2) and
α-(1→6) linkages obtained from Candida kefyr IFO 0586
strain. Carbohydr. Res..

[46] Willför S., Sjöholm R., Laine C., Holmbom B. (2002). Structural features
of water-soluble arabinogalactans from Norway spruce and Scots pine
heartwood. Wood Sci. Technol..

[47] Komura D. L., Ruthes A. C., Carbonero E. R., Alquini G., Rosa M. C. C., Sassaki G. L., Iacomini M. (2010). The origin
of mannans found in submerged
culture of basidiomycetes. Carbohydr. Polym..

[48] Cipriani T. R., Mellinger C. G., Bertolini M. L. C., Baggio C. H., Freitas C. S., Marques M. C. A., Gorin P. A. J., Sassaki G. L., Iacomini M. (2009). Gastroprotective
effect of a type I arabinogalactan from soybean meal. Food Chem..

[49] Cipriani T. R., Mellinger C. G., De Souza L. M., Baggio C. H., Freitas C. S., Marques M. C., Gorin P. A. J., Sassaki G. L., Iacomini M. (2006). A polysaccharide
from a tea (infusion) of *Maytenus ilicifolia* leaves
with anti-ulcer protective effects. J. Nat.
Prod..

[50] Delgobo C.
L., Gorin P. A. J., Tischer C. A., Iacomini M. (1999). The free reducing oligosaccharides
of angico branco (*Anadenanthera colubrina*) gum exudate:
an aid for structural assignments in the heteropolysaccharide. Carbohydr. Res..

[51] Fransen C. T. M., Haseley S. R., Huisman M. M. H., Schols H. A., Voragen A. G. J., Kamerling J. P., Vliegenthart J. F. G. (2000). Studies
on the structure of a lithium-treated
soybean pectin: characteristics of the fragments and determination
of the carbohydrate substituents of galacturonic acid. Carbohydr. Res..

[52] Gorin P. A. J., Mazurek M. (1975). Further studies on
the assignment of signals in ^13^C magnetic resonance spectra
of aldoses and derived methyl
glycosides. Can. J. Chem..

[53] Renard C. M. G. C., Lahaye M., Mutter M., Voragen F. G. J., Thibault J. F. (1997). Isolation
and structural characterisation of rhamnogalacturonan oligomers generated
by controlled acid hydrolysis of sugar-beet pulp. Carbohydr. Res..

[54] Tischer C. A., Gorin P. A. J., Iacomini M. (2002). The free reducing oligosaccharides
of gum arabic: aids for structural assignments in the polysaccharide. Carbohydr. Polym..

[55] Brecker L., Wicklein D., Moll H., Fuchs E. C., Becker W. M., Petersen A. (2005). Structural and immunological properties
of arabinogalactan
polysaccharides from pollen of timothy grass (*Phleum pratense* L.). Carbohydr. Res..

[56] Makarova E. N., Shakhmatov E. G., Belyy V. A. (2016). Structural characteristics of oxalate-soluble
polysaccharides of Sosnowsky’s hogweed (*Heracleumsosnowskyi
Manden*). Carbohydr. Polym..

[57] Shakhmatov E. G., Atukmaev K. V., Makarova E. N. (2016). Structural characteristics
of pectic
polysaccharides and arabinogalactan proteins from *Heracleum
sosnowskyi* Manden. Carbohydr. Polym..

[58] Yang B., Prasad K. N., Jiang Y. (2016). Structure
identification of a polysaccharide
purified from litchi (*Litchi chinensis* Sonn.) pulp. Carbohydr. Polym..

[59] Ovodova R. G., Golovchenko V. V., Popov S. V., Popova G. Y., Paderin N. M., Shashkov A. S., Ovodov Y. S. (2009). Chemical composition and anti-inflammatory
activity of pectic polysaccharide isolated from celery stalks. Food Chem..

[60] Popov S. V., Ovodova R. G., Golovchenko V. V., Popova G. Y., Viatyasev F. V., Shashkov A. S., Ovodov Y. S. (2011). Chemical
composition and anti-inflammatory
activity of a pectic polysaccharide isolated from sweet pepper using
a simulated gastric medium. Food Chem..

[61] Nascimento G. E., Iacomini M., Cordeiro L. M. C. (2017). New
findings on green sweet pepper
(*Capsicum annum*) pectins: Rhamnogalacturonan and
type I and II arabinogalactans. Carbohydr. Polym..

[62] Harholt J., Suttangkakul A., Vibe Scheller H. (2010). Biosynthesis of Pectin. Plant
Physiol..

[63] Messana I., Cabras T., Pisano E., Sanna M. T., Olianas A., Manconi B., Pellegrini M., Paludetti G., Scarano E., Fiorita A., Agostino S., Contucci A. M., Calo L., Picciotti P. M., Manni A., Bennick A., Vitali A., Fanali C., Inzitari R., Castagnola M. (2008). Trafficking
and Post secretory Events Responsible for the Formation of Secreted
Human Salivary Peptides. Mol. Cell. Proteomics.

[64] Gardner A., Parkes H. G., Carpenter G. H., So P. W. (2018). Developing and standardising
a protocol for quantitative proton nuclear magnetic resonance (^1^H-NMR) Spectroscopy of Saliva. J. Proteome
Res..

[65] Cabras T., Pisano E., Mastinu A., Denotti G., Pusceddu P. P., Inzitari R., Fanali C., Nemolato S., Castagnola M., Messana I. (2010). Alterations of the
Salivary Secretory Peptidome Profile
in Children Affected by Type 1 Diabetes. Mol.
Cell. Proteomics.

[66] Castagnola M., Cabras T., Iavarone F., Vincenzoni F., Vitali A., Pisano E., Nemolato S., Scarano E., Fiorita A., Vento G., Tirone C., Romagnoli C., Cordaro M., Paludetti G., Faa G., Messana M. (2012). Top-down platform
for deciphering the human salivary proteome. J. Matern. Fetal Neonatal Med..

[67] Padiglia A., Orrù R., Boroumand M., Olianas A., Manconi B., Sanna M. T., Desiderio C., Iavarone F., Liori B., Messana I., Castagnola M., Cabras T. (2018). Extensive characterization
of the human salivary basic Proline-rich proteins family by top-down
mass spectrometry. J. Proteome Res..

[68] Prodan A., Brand H., Imangaliyev S., Tsivtsivadze E., Van Der Weijden F., de Jong A., Paauw A., Crielaard W., Keijser B., Veerman E. (2016). A Study of the Variation
in the Salivary
Peptide Profiles of Young Healthy Adults Acquired Using MALDI-TOF
MS. PLoS One.

[69] Huq N. L., Cross K. J., Ung M., Myroforidis H., Veith P. D., Chen D., Stanton D., He H., Ward B. R., Reynolds E. C. (2007). A Review of the Salivary Proteome
and Peptidome and Saliva-derived Peptide Therapeutics. Int. J. Pept. Res. Ther..

[70] Arthur, R. A. ; Hashizume, L. N. ; Henz, S. L . Tópicos em Bioquímica e Microbiologia Bucais [Topics in Oral Biochemistry and Microbiology]. In Desenvolvimento da Lesão de Cárie [Development of the Caries Lesion]. 2nd ed.; Henz, S. L. ; Hashizume, L. N. ; Arthur, R. A. , Eds., Editora da UFRGS: Porto Alegre, RS, Brazil, 2021, p 179−186.

[71] Wang K., Zhou X., Li W., Zhang L. (2019). Human salivary proteins
and their peptidomimetics: Values of function, early diagnosis, and
therapeutic potential in combating dental caries. Arch. Oral Biol..

[72] Inzitari R., Cabras T., Rossetti D. V., Fanali C., Vitali A., Pellegrini M., Paludetti G., Manni A., Giardina B., Messana I., Castagnola M. (2006). Detection in human saliva of different
statherin and P-B fragments and derivatives. Proteomics.

[73] Watrelot A. A., Schulz D. L., Kennedy J. A. (2017). Wine polysaccharides influence tannin-protein
interactions. Food Hydrocolloids.

[74] García-Estévez I., Pérez-Gregorio R., Soares S., Mateus N., De Freitas V. (2017). Oenological
perspective of red wine astringency. OENO One.

[75] Soares S., García-Estévez I., Ferrer-Galego R., Brás N. F., Brandão E., Silva M., Teixeira N., Fonseca F., Sousa S. F., Ferreira-da-Silva F., Mateus N., de Freitas V. (2018). Study of human
salivary proline-rich
proteins interaction with food tannins. Food
Chem..

[76] Quijada-Morín N., Williams P., Rivas-Gonzalo J. C., Doco T., Escribano-Bailón M. T. (2014). Polyphenolic,
polysaccharide and oligosaccharide composition of Tempranillo red
wines and their relationship with the perceived astringency. Food Chem..

[77] Le
Bourvellec C., Bouchet B., Renard C. M. G. C. (2005). Non-covalent
interaction between procyanidins and apple cell wall material. Part
III: Study on model polysaccharides. Biochim.
Biophys. Acta Gen. Subj..

[78] Watrelot A. A., Renard C. M. G. C., Le Bourvellec C. (2015). Comparison of microcalorimetry and
haze formation to quantify the association of B-type procyanidins
to poly-l-proline and bovine serum albumin. LWT--Food Sci. Technol..

[79] Torres-Rochera B., Manjón E., Escribano-Bailón M. T., García-Estévez I. (2023). Role of Anthocyanins
in the Interaction between Salivary Mucins and Wine Astringent Compounds. Foods.

[80] Brown F. N., Mackie A. R., He Q., Branch A., Sarkar A. (2021). Protein–saliva
interactions: a systematic review. Food Funct..

[81] Jahmidi-Azizi N., Oliva R., Winter R. (2023). Alcohol-induced Conformational Changes
and Thermodynamic Signatures in the Binding of Polyphenols to Proline-rich
Salivary Proteins. Chem—Eur. J..

[82] Dufourc E. J. (2024). Wine tannins,
saliva proteins and membrane lipids: A review and perspectives for
neurodegenerative diseases. Biophys. Chem..

[83] Tortorella A., Petraccone L., Del Vecchio P., Oliva R., Winter R. (2026). Fatty Acid-Induced
Modulation of Saliva Protein-Polyphenol Interactions: Molecular Signatures
Underlying the Camembert Effect of Astringency. Biomacromolecules.

[84] Anderson, C. T. . Pectic Polysaccharides in Plants: Structure, Biosynthesis, Functions, and Applications. In: Cohen, E. ; Merzendorfer, H. , Eds. Extracellular Sugar-Based Biopolymers Matrices. Biologically-Inspired Systems, vol 12. Springer, Cham, 2019. DOI: 10.1007/978-3-030-12919-4_12.

[85] Robledo, V. R. , Vázquez, L. I. C. . In: Masuelli, M. , Ed., Pectins - Extraction, Purification, Characterization and Applications, 2020. IntechOpen. DOI: 10.5772/intechopen.85588.

