# Supplementary material for: Impact of Proline-Rich Salivary Proteins on Interactions between Catechin and Polysaccharides
Source: J Agric Food Chem. 2026 Aug 28;74(35):27890–903. doi: 10.1021/acs.jafc.6c07975 (PMC13573559; doi:10.1021/acs.jafc.6c07975)
Supplement: Supplementary file 1 [file jf6c07975_si_001.pdf]

# IMPACT OF PROLINE-RICH SALIVARY PROTEINS ON INTERACTIONS BETWEEN CATECHIN AND POLYSACCHARIDES

Philippe Rodrigues Benedetti[a], Leociley Rocha Alencar Menezes[a], Guilherme Lanzi Sassaki[a*]

[a] Department of Biochemistry and Molecular Biology, Federal University of Paraná, Curitiba, Paraná, 81.531-980, Brazil.

* Corresponding author e-mail: sassaki@ufpr.br.

## SUPPLEMENTARY MATERIAL

**SUPPLEMENTARY FIGURE S1 -** ELUTION PROFILES OF SOLUBLE POLYSACCHARIDE FRACTIONS DETERMINED BY HPSEC USING A REFRACTIVE INDEX DETECTOR.

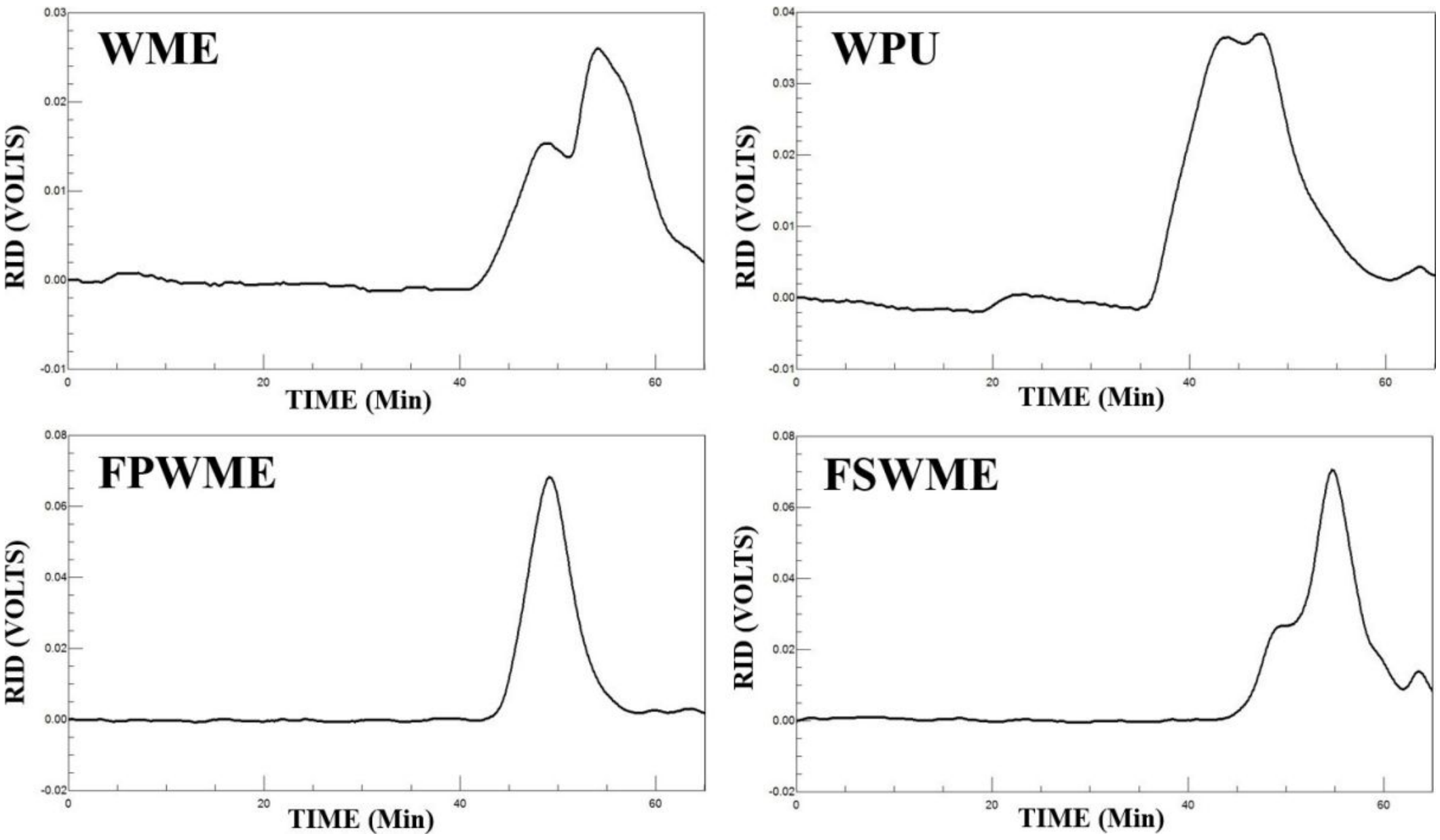


**Legend:** Elution profile of the samples: water-soluble polysaccharide from Merlot wine (WME); water-soluble polysaccharide from grape pulp (WPU); Fehling precipitate from the WME fraction; and Fehling supernatant from the WME fraction. The Y-axis represents the voltage recorded by the refractive index detector (RID), and the X-axis represents the elution time.

**SUPPLEMENTARY FIGURE S2 –** NMR SPECTRUM (1D) – $^{1}H$ AND $^{13}C$ OF THE SOLUBLE FRACTION OF MERLOT WINE (WME).

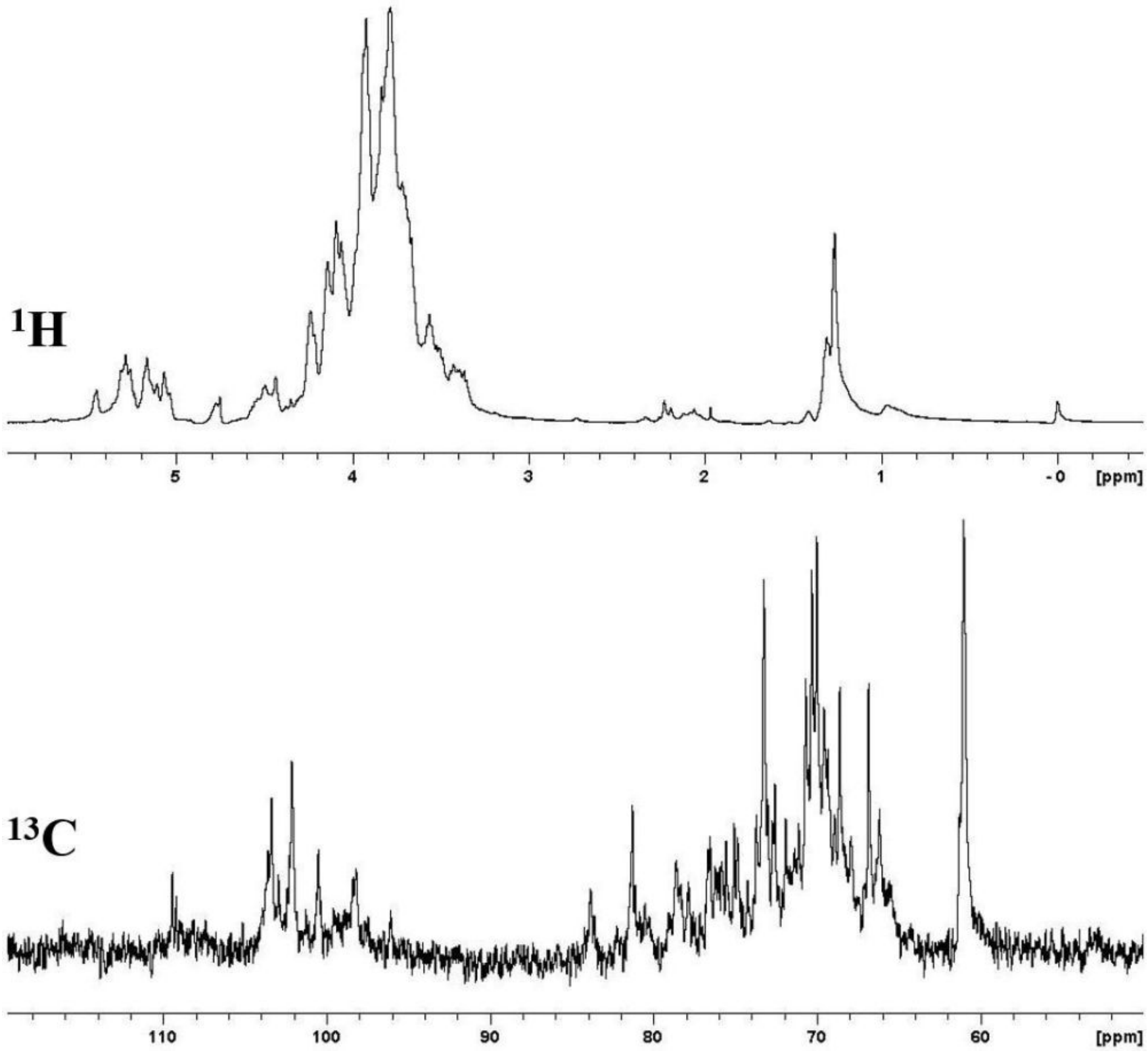


**Legend:** $^{1}H$ NMR spectrum (upper) and $^{13}C$ NMR spectrum (lower) of the water-soluble polysaccharide fraction from Merlot wine. Signal overlap can be observed in both spectra due to the complex mixture of polysaccharides in this fraction. $^{1}H$: 600 MHz, $^{13}C$: 150 MHz, 30 ºC, $D_2O$.

**SUPPLEMENTARY FIGURE S3 –** NMR SPECTRUM (2D) – HSQC OF THE FEHLING SUPERNATANT FRACTION OF WINE.

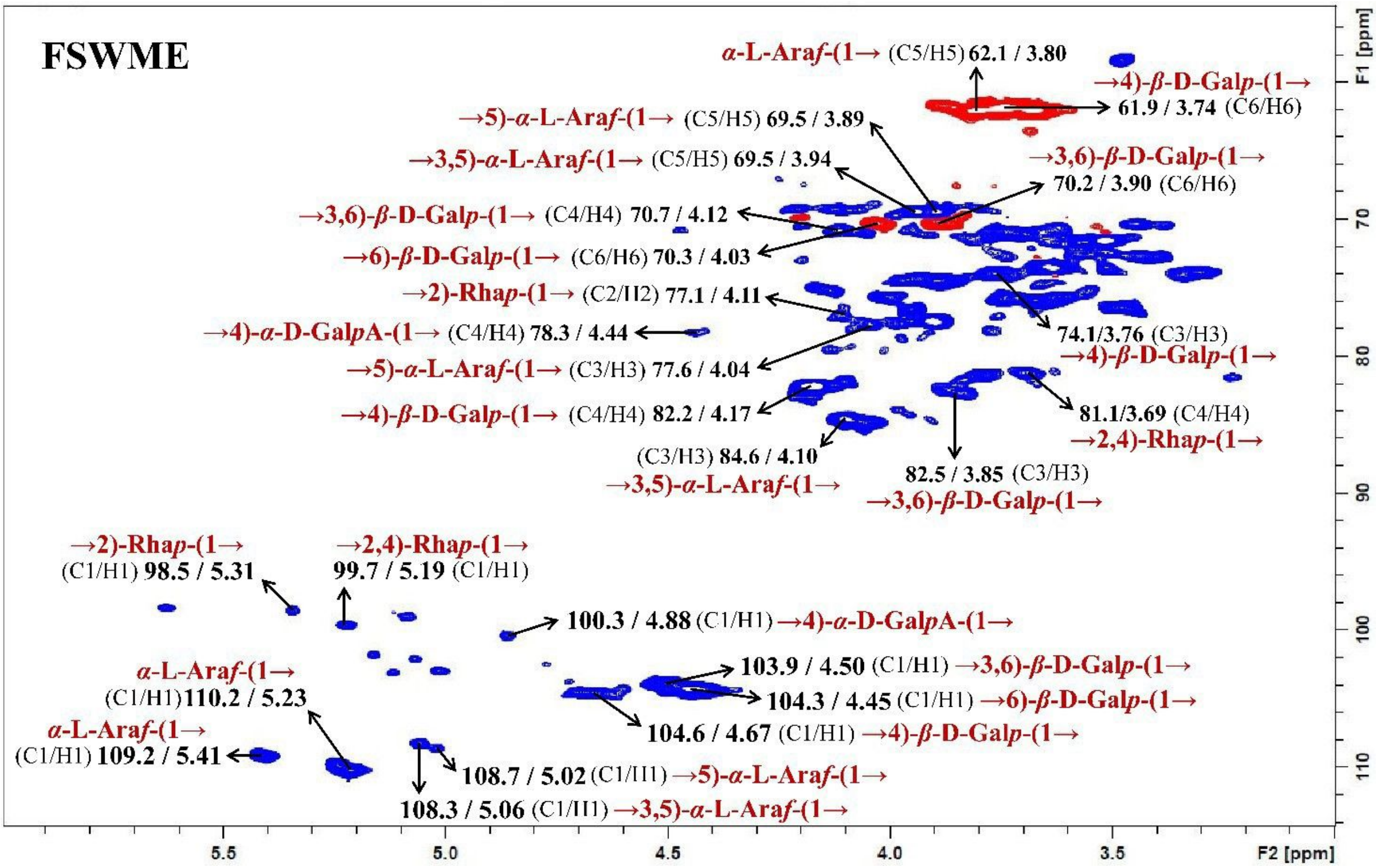


**Legend:** Chemical shifts are expressed in ppm in the format ($^{13}$C/$^{1}$H), with the corresponding carbon and hydrogen atoms indicated after the shifts, followed by the structure identification (in red). $^{1}$H: 600 MHz, $^{13}$C: 150 MHz, 30 ºC, $D_2O$. FSWME: Fehling supernatant from the soluble fraction of Merlot wine.

The $^{1}$H-$^{13}$C HSQC NMR analysis of the FSWME fraction (Supplementary Figure S3) revealed chemical shifts compatible with the signals of anomeric systems (C1/H1) at 103.9/4.50, 104.3/4.45, and 104.6/4.67 ppm, indicating the presence of the units →3,6)-β-D-Gal*p*-(1→, 6)-β-D-Gal*p*-(1→, and →4)-β-D-Gal*p*-(1→, respectively. The signals at 82.5/3.85 (C3/H3) and 70.2/3.90 ppm (C6/H6) corroborate the presence of the residue →3,6)-β-D-Gal*p*-(1→, while the signal at 82.2/4.17 ppm (C4/H4) confirms the presence of the residue →4)-β-D-Gal*p*-(1→. The signals at 109.2/5.41 (C1/H1), 110.2/5.23 (C1/H1), and 62.1/3.80 ppm (C5/H5) are typical of non-reducing terminals of α-L-Ara*f*. Thus, the presence of type I AG is suggested, with a main chain of β-D-Gal*p* linked (1→4) and substituted by α-L-Ara*f* units, and type II AG, with a main chain of β-D-Gal*p* linked (1→3)/(1→6), with branches of Gal or Ara at 3-*O* or 6-*O*.[1–12]

The presence of pectins is confirmed by the signal (C1/H1) at 100.3/4.88 ppm, indicating the presence of the units →4)-α-D-Gal*p*A-(1→. The signal at 78.3/4.44 ppm (C4/H4) corroborates the presence of the residue →4)-α-D-Gal*p*A-(1→. The signals at 99.7/5.19 ppm, 98.5/3.31 ppm (C1/H1) indicate the existence of units of →2,4)-Rha*p*-(1→, and →2)-Rha*p*-(1→. The signals at 77.1/4.11 (C2/H2) and 81.1/3.69 ppm (C4/H4) corroborate the presence of the residues →2)-Rha*p*-(1→ and →2,4)-Rha*p*-(1→. Therefore, it is possible to suggest the presence of type I RG, formed by a main

chain of intercalated α-D-Gal*p*A and Rha*p* units. Additionally, this type I RG may present arabinan branches, due to the presence of signals (C1/H1) at 108.3/5.06 and 108.7/5.02 ppm, indicating the existence of units of →3,5)-α-L-Ara*f*-(1→ and →5)-α-L-Ara*f*-(1→. The signals at 84.6/4.10 (C3/H3) and 69.5/3.89 ppm (C5/H5) corroborate the presence of the residues →3,5)-α-L-Ara*f*-(1→ and →5)-α-L-Ara*f*-(1→.[6,8–14]

The methylation analysis of the FSWME fraction (Supplementary Table S1) corroborated the monosaccharide composition and the $^{1}H$-$^{13}C$ HSQC NMR analysis. The results evidenced the presence of AG I, due to the existence of the derivative →4)-Gal*p*-(1→ (2,3,6-$Me_3$), which composes the main chain of this polysaccharide. Additionally, the presence of →3,6)-Gal*p*-(1→ (2,4-$Me_2$) and →6)-Gal*p*-(1→ (2,3,4-$Me_3$) corroborates the existence of AG II, and the terminals of Ara*f*-(1→ (2,3,5-$Me_3$) are present in both polysaccharides.[1–3,7] The existence of Rha*p*-(1→ (2,3,4-$Me_3$), →2)-Rha*p*-(1→ (3,4-$Me_2$), and →2,4)-Rha*p*-(1→ (3-Me) suggests the presence of RG I, along with the increase of the derivative →4)-Gal*p*-(1→ (2,3,6-$Me_3$) after the carboxy-reduction procedure, indicating the presence of →4)-Gal*p*A-(1→. The derivative →2,4)-Rha*p*-(1→ indicates the presence of branches in the main chain. These branches are composed of →5)-Ara*f*-(1→ (2,3-$Me_2$) and →3,5)-Ara*f*-(1→ (2-Me). Additionally, the presence of the derivatives →3,4)-Gal*p*-(1→ and →2,4)-Gal*p*-(1→ after carboxy- reduction suggests the presence of RG II, indicating a main chain of GalA linked 1→4, with branches at 2 or 3-*O*.[15,16]

**SUPPLEMENTARY TABLE S1 –** ALDITOL ACETATE DERIVATIVES, PARTIALLY METHYLATED AND CARBOXY-REDUCED PARTIALLY METHYLATED, AND GLYCOSIDIC LINKAGES OF THE FSWME FRACTION.

| RT (min) | *O*-Me-Alditol acetate | Native (%) | Carboxy-reduced* (%) | Structure |
|---|---|---|---|---|
| 20.58 | 2,3,5-$Me_3$ | 21.91 | 2.97 | Ara*f*-(1 → |
| 21.45 | 2,3,4-$Me_3$ | 1.28 | 1.12 | Rha*p*-(1 → |
| 22.80 | 2-Me | 1.21 | 0.82 | → 3,4)-Rha*p*-(1 → |
| 23.50 | 3,4-$Me_2$ | - | 3.47 | → 2)-Rha*p*-(1 → |
| 23.56 | 2,3-$Me_2$ | 9.89 | 8.69 | → 5)-Ara*f*-(1 → |
| 24.41 | 2,3,4,6-$Me_3$ | 5.28 | 9.42 | Glc*p*-(1 → |
| 24.50 | 2,3,4,6-$Me_3$ | 5.86 | 5.97 | Man*p*-(1 → |
| 24.95 | 2,3,4,6-$Me_3$ | 9.57 | 17.97 | Gal*p*-(1 → |
| 25.27 | 2-Me | 3.43 | 3.64 | → 3,5)-Ara*f*-(1 → |
| 25.51 | 3-Me | 1.21 | 1.52 | → 2,4)-Rha*p*-(1 → |
| 26.29 | 3,4,6-$Me_3$ | 5.37 | 6.93 | → 2)-Gal*p*-(1 → |
| 26.41 | 2,3,6-$Me_3$ | 3.00 | 4.08 | → 4)-Gal*p*-(1 → |
| 27.65 | 2,3,4-$Me_3$ | 11.72 | 11.66 | → 6)-Gal*p*-(1 → |

| | | | | |
|---|---|---|---|---|
| 27.81 | 2,6-$Me_2$ | - | 2.14 | → 3,4)-Gal*p*-(1 → |
| 28.21 | 3,6-$Me_2$ | - | 0.84 | → 2,4)-Gal*p*-(1 → |
| 28.81 | 3,4-$Me_2$ | 6.43 | 4.31 | → 2,6)-Gal*p*-(1 → |
| 29.43 | 2,4-$Me_2$ | 10.13 | 10.66 | → 3,6)-Gal*p*-(1 → |
| 30.05 | 2-Me | 3.71 | 3.79 | → 3,4,6)-Gal*p*-(1 → |

**Legend:** The table presents the partially methylated alditol acetate derivatives obtained after methylation and derivatization. The identified and suggested structure for each derivative is shown, as well as the percentage of each one before and after carboxy-reduction. The samples were (*) carboxy-reduced using the carbodiimide method (TAYLOR & CONRAD[17]) prior to methylation analysis. RT: retention time; Me: methyl; Ara: arabinose; Rha: rhamnose; Glc: glucose; Man: mannose; Gal: galactose; *p*: pyranosidic conformation; *f*: furanosidic conformation.

**SUPPLEMENTARY TABLE S2 –** FRAGMENTATION PROFILE OF PARTIALLY METHYLATED ALDITOL ACETATE DERIVATIVES AND THEIR GLYCOSIDIC LINKAGES OF THE FPWME FRACTION.

| RT (min) | Fragments (*m/z*) | *O*-Me-alditol acetate | % | Structure |
|---|---|---|---|---|
| 24.510 | 87, 102, 118, 129, 161 | 2,3,4,6-*O*-$Me_4$ | 45.61 | Man*p*-(1 → |
| 26.327 | 87, 88, 129, 130, 161 | 3,4,6-*O*-$Me_3$ | 11.24 | → 2)-Man*p*-(1 → |
| 27.081 | 87, 99, 102, 118, 129 | 2,3,4-*O*-$Me_3$ | 3.80 | → 6)-Man*p*-(1 → |
| 28.873 | 87, 88, 129, 130 | 3,4-*O*-$Me_2$ | 39.35 | → 2,6)-Man*p*-(1 → |

**Legend:** The table presents the partially methylated alditol acetate derivatives obtained after methylation and derivatization. The identified and suggested structure for each derivative is shown, along with its percentage and the main fragments (*m/z*) obtained from gas chromatography–mass spectrometry (GC-MS) analysis. RT: retention time; Me: methyl; Man: mannose; *p*: pyranosidic conformation.

**SUPPLEMENTARY TABLE S3 –** PARTIALLY METHYLATED AND CARBOXY-REDUCED PARTIALLY METHYLATED ALDITOL ACETATE DERIVATIVES AND THEIR GLYCOSIDIC LINKAGES FOUND IN THE WPU FRACTION.

| RT (min) | *O*-Me-Alditol Acetate | Native (%) | Carboxy-reduced* (%) | Structure |
|---|---|---|---|---|
| 20.543 | 2,3,5-$Me_3$ | 22.73 | 8.83 | Ara*f*-(1 → |
| 21.373 | 2,3,4-$Me_3$ | 0.59 | 1.66 | Rha*p*-(1 → |
| 23.442 | 3,4-$Me_2$ | 7.27 | 10.05 | → 2,6)-Gal*p*-(1 → |
| 23.530 | 2,3-$Me_2$ | 29.92 | 5.78 | → 5)-Ara*f*-(1 → |
| 24.344 | 2,3,4,6-$Me_4$ | 1.33 | 5.98 | Glc*p*-(1 → |
| 24.436 | 2,3,4,6-$Me_4$ | 1.17 | - | Man*p*-(1 → |
| 24.887 | 2,3,4,6-$Me_4$ | 9.71 | 11.65 | Gal*p*-(1 → |
| 25.234 | 2-Me | 8.48 | 2.37 | → 3,5)-Ara*f*-(1 → |
| 25.451 | 3-Me | 2.50 | 3.67 | → 2,4)-Rha*p*-(1 → |
| 26.468 | 2,3,6-$Me_3$ | 2.27 | 34.63 | → 4)-Gal*p*-(1 → |
| 26.762 | 2,4,6-$Me_3$ | 1.73 | 2.09 | → 3)-Gal*p*-(1 → |

| | | | | |
|---|---|---|---|---|
| 27.582 | 2,3,4-$Me_3$ | 4.63 | 3.97 | → 6)-Gal*p*-(1 → |
| 27.707 | 2,6-$Me_2$ | - | 1.82 | → 3,4)-Gal*p*-(1 → |
| 28.626 | 2,3-$Me_2$ | 1.84 | 1.15 | → 4,6)-Gal*p*-(1 → |
| 29.362 | 2,4-$Me_2$ | 4.86 | 5.30 | → 3,6)-Gal*p*-(1 → |
| 29.998 | 2-Me | 0.99 | 1.05 | → 3,4,6)-Gal*p*-(1 → |

**Legend:** The table presents the partially methylated alditol acetate derivatives obtained after methylation and derivatization. The identified and suggested structure for each derivative is shown, as well as the percentage of each one before and after carboxy-reduction. The samples were (*) carboxy-reduced using the carbodiimide method (TAYLOR & CONRAD[17]) prior to methylation analysis. RT: retention time; Me: methyl; Ara: arabinose; Rha: rhamnose; Glc: glucose; Man: mannose; Gal: galactose; *p*: pyranosidic conformation; *f*: furanosidic conformation.

**SUPPLEMENTARY FIGURE S4** – $^1$H NMR SPECTRUM OF THE SUPERNATANT FRACTION IN TFA (0.1%).

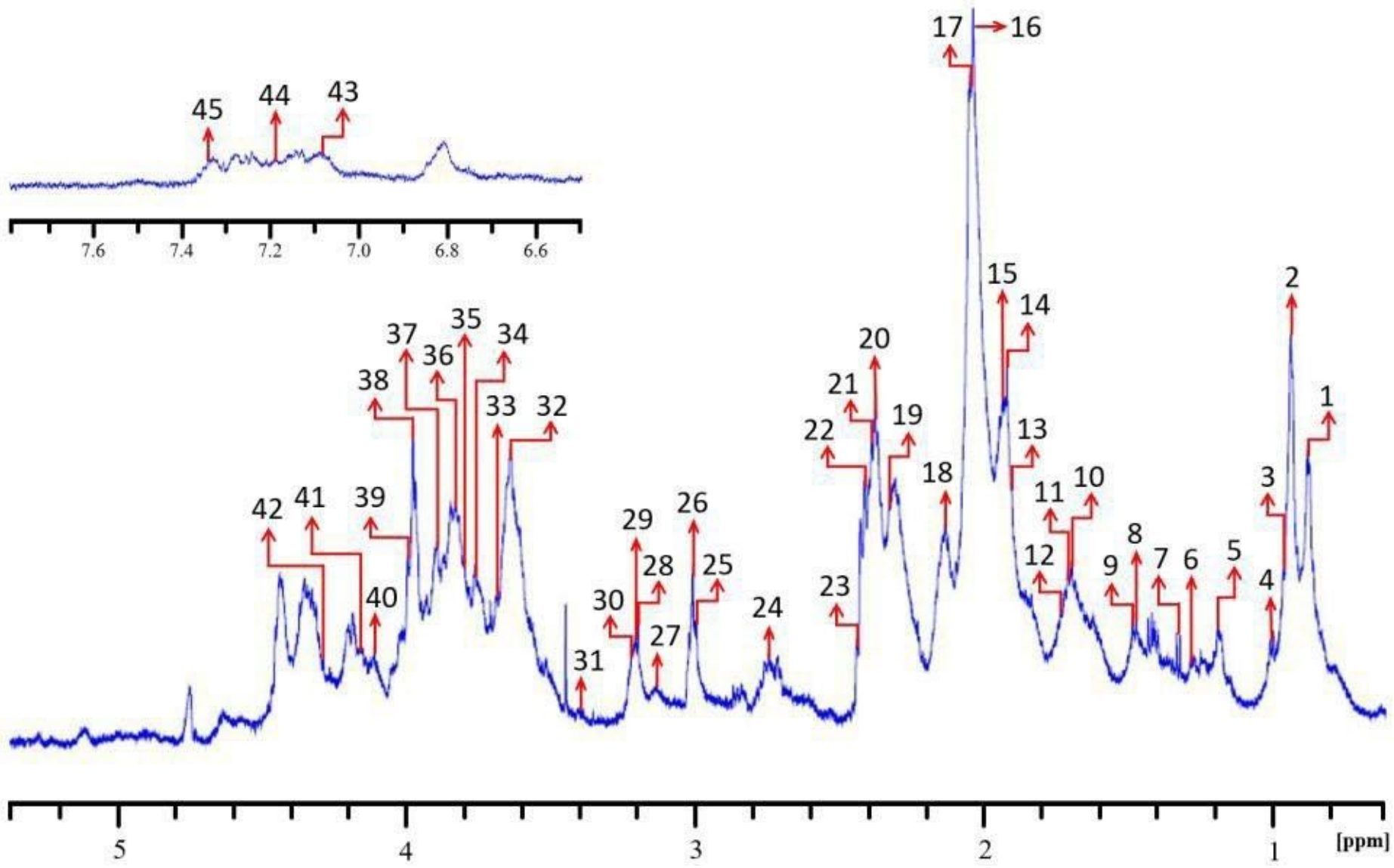


**Legend:** $^1$H spectrum acquired at 600 MHz, 30 °C, in $D_2O$. Numbers indicate identified compounds (see Supplementary Table S4).

**SUPPLEMENTARY TABLE S4** – COMPOUNDS IDENTIFIED IN THE ACIDIC SUPERNATANT FRACTION (TFA 0.1%) BY $^1$H NMR (SEE SUPPLEMENTARY FIGURE S4).

| Nº | $^1$H (ppm) | Compound | Nº | $^1$H (ppm) | Compound |
|---|---|---|---|---|---|
| 1 | 0.88 | Fatty Acid | 24 | 2.76 | Aspartate |
| 2 | 0.94 | Isoleucine | 25 | 3.00 | Tyrosine |
| 3 | 0.96 | Leucine | 26 | 3.01 | Lysine |
| 4 | 1.01 | Isoleucine | 27 | 3.14 | Histidine |
| 5 | 1.18 | Fatty Acid | 28 | 3.20 | Phosphoethanolamine |
| 6 | 1.28 | Fatty Acid | 29 | 3.21 | Choline |
| 7 | 1.32 | Lactate | 30 | 3.22 | Taurine |
| 8 | 1.47 | Isoleucine | 31 | 3.40 | Proline |
| 9 | 1.48 | Alanine | 32 | 3.64 | Valine |

| | | | | | |
|---|---|---|---|---|---|
| 10 | 1.70 | Leucine | 33 | 3.69 | Isoleucine |
| 11 | 1.71 | Arginine | 34 | 3.76 | Lysine |
| 12 | 1.73 | Lysine | 35 | 3.78 | Arginine |
| 13 | 1.90 | Lysine | 36 | 3.83 | Tyrosine |
| 14 | 1.92 | Acetate | 37 | 3.90 | Aspartate |
| 15 | 1.93 | Arginine | 38 | 3.98 | Histidine |
| 16 | 2.04 | *N*-acetyl | 39 | 4.00 | Phenylalanine |
| 17 | 2.05 | Proline | 40 | 4.11 | Lactate |
| 18 | 2.14 | Glutamine | 41 | 4.15 | Proline |
| 19 | 2.34 | Proline | 42 | 4.29 | Threonine |
| 20 | 2.37 | Glutamate | 43 | 7.09 | Histidine |
| 21 | 2.39 | Pyruvate | 44 | 7.19 | Tyrosine |
| 22 | 2.41 | Succinate | 45 | 7.34 | Phenylalanine |
| 23 | 2.44 | Glutamine | | | |

**Legend:** The numbers in the table correspond to those shown in the previous figure (Supplementary Figure S4). They indicate the compounds identified based on their chemical shifts (ppm), obtained through $^{1}H$ NMR and compared with the references: Berthan *et al.*[18], Neyraud *et al.*[19], Gardner *et al.*[20], Silwood *et al.*[21], Takeda *et al.*[22]

**SUPPLEMENTARY FIGURE S5 -** CHROMATOGRAMS OF SUPERNATANT FRACTIONS (0.1% TFA), OBTAINED USING ÄKTA SYSTEM WITH HITRAP Q HP ANION EXCHANGE COLUMN.

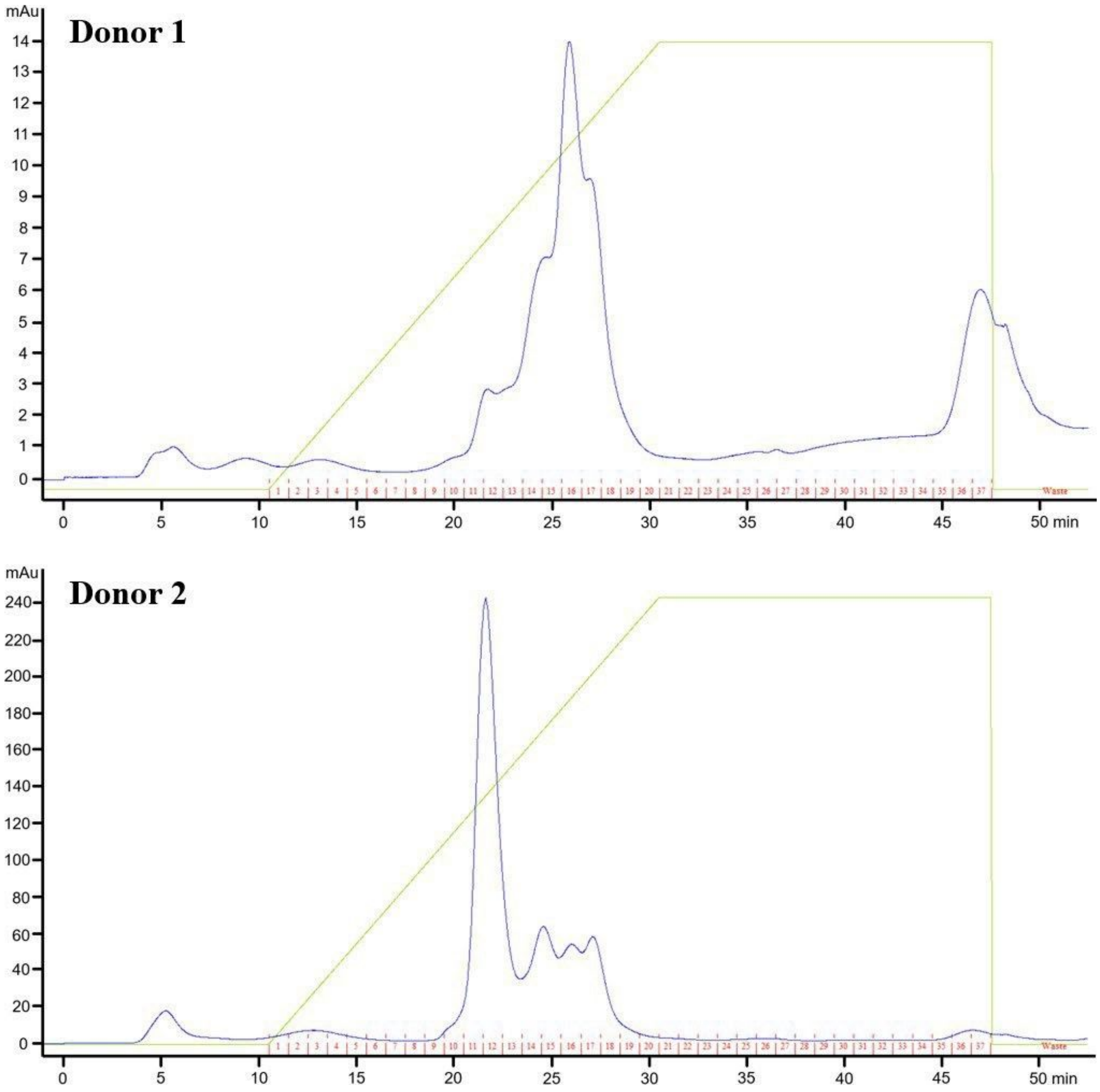


**Legend:** Chromatograms obtained by liquid chromatography with UV detection (blue line) of two protein samples derived from the saliva of two donors (donor 1 – upper, and donor 2 – lower). The Y-axis shows the UV detection intensity, and the X-axis shows the time in minutes. Red subdivisions indicate the fractions collected using an automatic fraction collector. The green line represents the injection of buffer B, starting at 10 minutes and reaching maximum concentration at 30 minutes.

## REFERENCES

[1] Cipriani, T. R., Mellinger, C. G., Bertolini, M. L. C., Baggio, C. H., Freitas, C. S., Marques, M. C. A., & Iacomini, M. (2009). Gastroprotective effect of a type I arabinogalactan from soybean meal. *Food Chemistry*, 115 (2), 687-690. https://doi.org/10.1016/j.foodchem.2008.12.017.

[2] Cipriani, T. R., Mellinger, C. G., De Souza, L. M., Baggio, C. H., Freitas, C. S., Marques, M. C., & Iacomini, M. (2006). A polysaccharide from a tea (infusion) of *Maytenus ilicifolia* leaves with anti-ulcer protective effects. *Journal of Natural Products*, 69 (7), 1018-1021. https://doi.org/10.1021/np060045z.

[3] Delgobo, C. L., Gorin, P. A. J., Tischer, C. A., & Iacomini, M. (1999). The free reducing oligosaccharides of angico branco (*Anadenanthera colubrina*) gum exudate: an aid for structural assignments in the heteropolysaccharide. *Carbohydrate Research*, 320, (3-4), 167-175. https://doi.org/10.1016/s00086215(99)00159-7.

[4] Fransen, C. T. M., Haseley, S. R., Huisman, M. M. H., Schols, H. A., Voragen, A. G. J., Kamerling, J. P., & Vliegenthart, J. F. G. (2000). Studies on the structure of a lithium-treated soybean pectin: characteristics of the fragments and determination of the carbohydrate substituents of galacturonic acid. *Carbohydrate Research*, 328, 539-547. https://doi.org/10.1016/S0008-6215(00)00130-0.

[5] Gorin, P. A. J., & Mazurek, M. (1975). Further studies on the assignment of signals in $^{13}C$ magnetic resonance spectra of aldoses and derived methyl glycosides. *Canadian Journal of Chemistry*, 53, 1212-1222. https://doi.org/10.1139/v75-168.

[6] Renard, C. M. G. C., Lahaye, M., Mutter, M., Voragen, F. G. J., & Thibault, J. F. (1997). Isolation and structural characterisation of rhamnogalacturonan oligomers generated by controlled acid hydrolysis of sugar- beet pulp. *Carbohydrate Research*, 305 (2), 271-280. https://doi.org/10.1016/S0008-6215(97)10028-3.

[7] Tischer, C. A., Gorin, P. A. J., & Iacomini, M. (2002). The free reducing oligosaccharides of gum arabic: aids for structural assignments in the polysaccharide. *Carbohydrate Polymers*, 47 (2), 151-158. https://doi.org/10.1016/S0144-8617(01)00173-4.

[8] Brecker, L., Wicklein, D., Moll, H., Fuchs, E. C., Becker, W. M., & Petersen, A. (2005). Structural and immunological properties of arabinogalactan polysaccharides from pollen of timothy grass (*Phleum pratense* L.). *Carbohydrate Research*, 340, (4), 657-663. https://doi.org/10.1016/j.carres.2005.01.006.

[9] Makarova, E. N., Shakhmatov, E. G., & Belyy, V. A. (2016). Structural characteristics of oxalate- soluble polysaccharides of Sosnowsky's hogweed (*Heracleumsosnowskyi Manden*). *Carbohydrate Polymers*, 153, 66-77. https://doi.org/10.1016/j.carbpol.2016.07.089.

[10] Shakhmatov, E. G., Atukmaev, K. V. & Makarova, E. N. (2016). Structural characteristics of pectic polysaccharides and arabinogalactan proteins from *Heracleum sosnowskyi* Manden. *Carbohydrate Polymers*, 136, 1358-1369. https://doi.org/10.1016/j.carbpol.2015.10.041.

[11] Yang, B., Prasad, K. N., & Jiang, Y. (2016). Structure identification of a polysaccharide purified from litchi (*Litchi chinensis* Sonn.) pulp. *Carbohydrate Polymers*, 137, 570-575. https://doi.org/10.1016/j.carbpol.2015.10.088.

[12] Yuan, Y., Wang, Y. B., Jiang, Y., Prasad, K. N., Yang, J., Qu, H., Wang, Y., Jia, Y., Mo, H., & Yang, B. (2016). Structure identification of a polysaccharide purified from *Lycium barbarium* fruit. *International Journal of Biological Macromolecules*, 82, 696-701. https://doi.org/10.1016/j.ijbiomac.2015.10.069.

[13] Ovodova, R. G., Golovchenko, V. V., Popov, S. V., Popova, G. Y., Paderin, N. M., Shashkov, A. S., & Ovodov, Y. S. (2009). Chemical composition and anti-inflammatory activity of pectic polysaccharide isolated from celery stalks. *Food Chemistry*, 114 (2), 610-615. https://doi.org/10.1016/j.foodchem.2008.09.094.

[14] Popov, S. V., Ovodova, R. G., Golovchenko, V. V., Popova, G. Y., Viatyasev, F. V., Shashkov, A. S., & Ovodov, Y. S. (2011). Chemical composition and anti-inflammatory activity of a pectic polysaccharide isolated from sweet pepper using a simulated gastric medium. *Food Chemistry*, v. 124, p. 309-315. https://doi.org/10.1016/j.foodchem.2010.06.038.

[15] Harholt, J., Suttangkakul, A., & Scheller, H. V. (2010). Biosynthesis of Pectin. *Plant Physiology*, 153, 384-395. https://doi.org/10.1104/pp.110.156588.

[16] Nascimento, G. E., Iacomini, M., & Cordeiro, L. M. C. (2017). New findings on green sweet pepper (*Capsicum annum*) pectins: Rhamnogalacturonan and type I and II arabinogalactans. *Carbohydrate Polymers*, 171, 292-299. https://doi.org/10.1016/j.carbpol.2017.05.029.

[17] Taylor, R. L., & Conrad, H. E. (1972) Stoichiometric depolymerization of polyuronides and glycosaminoglycuronans to monosaccharides follow ingreduction of their carbodiimide-activated carboxyl group. *Biochemistry*, 11 (8), 1383-1388. https://doi.org/10.1021/bi00758a009.

[18] Bertram, H. C., Eggers, N., & Eller, N. (2009). Potential oh human saliva for nuclear magnetic resonance-based metabolomics and for health-related biomarker identification. *Analytical Chemistry*, 81, 9188-9193. https://doi.org/10.1021/ac9020598.

[19] Neyraund, E., Tremblay-Franco, M., Gregoire, S., Berdeaux, O., & Canlet, C. (2012). Relationships between the metabolome and the fatty acid composition of human saliva: effects of stimulation. *Metabolomics*, 9, 213-222. https://doi.org/10.1007/s11306-012-0440-6.

[20] Gardner, A., Parkes, H. G., Carpenter, G. H., & So, P. W. (2018). Developing and standardising a protocol for quantitative proton nuclear magnetic resonance ($^{1}$H-NMR) Spectroscopy of Saliva. *Journal of Proteome Research*, 17, 1521-1531. https://doi.org/10.1021/acs.jproteome.7b00847.

[21] Silwood, C. J. L., Lynch, E. J., Seddon, S., Sheerin, A., Claxson, A. D., & Grootveld, M. C. (1999). $^{1}$H-NMR analysis of microbial-derived organic acids in primary root carious lesions and saliva. *NMR in Biomedicine*, 12, 345-356. https://doi.org/10.1002/(sici)1099-1492(199910)12:6<345::aid-nbm580>3.0.co;2c.

[22] Takeda, I., Stretch, C., Barnaby, P., Bhatnager, K., Rankin, K., Fu, H., Weljie, A., Jha, N., & Slupsky, C. (2009). Understanding the human salivary metabolome. *NMR in Biomedicine*, 22, 577-584. https://doi.org/10.1002/nbm.1369.
